# Nutritional, Phytochemical, and Antioxidant Profiling of Cold-Pressed Oils and Press-Cake Byproducts from White, Yellow, and Blue Poppy (*Papaver somniferum* L.) Seeds

**DOI:** 10.1021/acsomega.6c09692

**Published:** 2026-09-30

**Authors:** Samiye Adal, Yunus Çetintaş, Merve Balaban, Ümit Çalışır, Gülşah Çalışkan Koç

**Affiliations:** † Department of Food Engineering, Faculty of Engineering, 53002 Afyon Kocatepe University, Afyonkarahisar 03200, Türkiye; ‡ Food Control, Research and Application Center, Afyon Kocatepe University, Afyonkarahisar 03200, Türkiye; § Food Quality Control and Analysis Program, Food Processing Department, Ula Ali Koçman Vocational School, 52986 Muğla Sıtkı Koçman University, Muğla 48640, Türkiye; ∥ Central Research Laboratories, Food Analysis Application and Research Center, Muğla Sıtkı Koçman University, Muğla 48000, Türkiye; ⊥ Science and Technology Application and Research Center, Kezer Campus, Siirt University, Siirt 56100, Türkiye; # Food Technology Program, Food Processing Department, Eşme Vocational School, 175652 Uşak University, Eşme, Uşak 64600, Türkiye

## Abstract

Seeds, cold-pressed oils (CPOs), and press-cake flours (PSCFs) derived from three poppy (*Papaver somniferum* L.) seed color varieties (white, yellow, and blue) were systematically characterized to elucidate compositional changes associated with the cold-pressing process. The effects of seed color, material type, and their interaction on the evaluated parameters were assessed using two-way analysis of variance. Relative to the whole seeds, the PSCFs were markedly richer in protein (33.70–37.45% versus 19.58–24.22%) and ash (12.38–13.46% versus 7.07–8.87%), lower in fat (9.72–10.59%), and were concentrated sources of Ca, Mg, P, K, Mn, Fe, and Zn, the white-seed flour ranking highest for most macroelements. Linoleic acid dominated every matrix (64.41–76.81% of total fatty acids), and the yellow-seed oil showed the highest unsaturated fraction (91.64%). The free amino acid pool was 33–97 times larger in the flours than in the seeds, and the yellow-seed flour was the richest source overall. The flours exhibited higher total phenolic content and stronger DPPH, FRAP, and CUPRAC activities than the seeds and oils, and the color × material interaction was significant for almost all variables (*p* < 0.05). Cold-pressed poppy press-cake flour is therefore a nutrient-dense, antioxidant-rich byproduct and a promising candidate for functional food applications, pending further safety, technological, and sensory evaluation.

## Graphical abstract

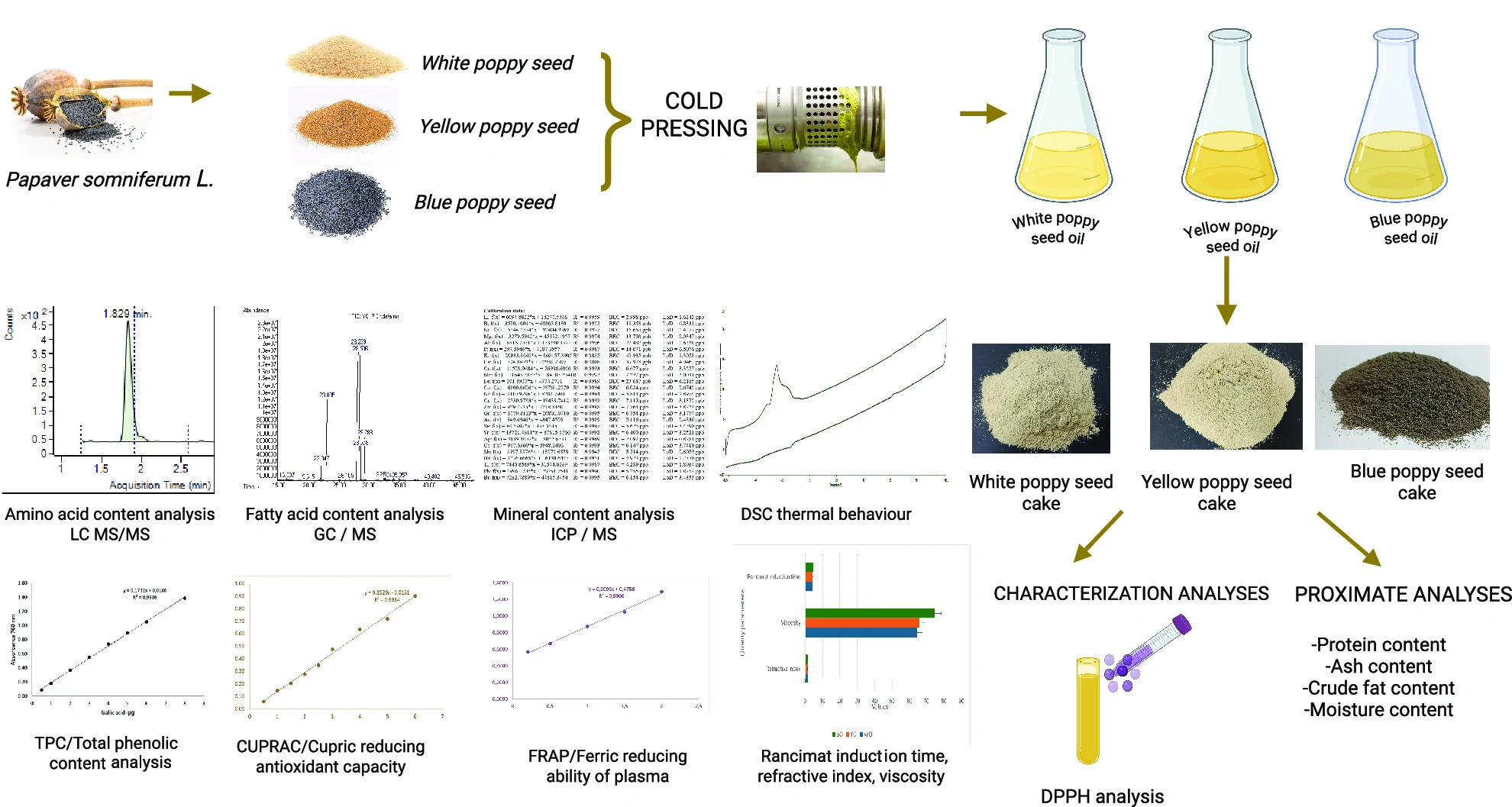

## Introduction

Poppy (*Papaver somniferum* L.) has long been valued for the analgesic alkaloids of its latex, but its seeds contain no opium and are used as food in their own right: they carry 28–54% oil and 20–30% protein, the oil being dominated by linoleic acid and other unsaturated fatty acids (UFAs).^1,2^ Cold-pressed poppy oil is distinguished by a nutty aroma and characteristic taste that suit it to salad dressings and to broader culinary and medicinal use.^3–5^ Cultivation favors hot summers and moderate rainfall, and in Türkiye, where the seed oil is widely used in cooking,^6^ production centers on Afyonkarahisar, Konya, Burdur, Isparta, and Kütahya;^7^ Afyonkarahisar poppy seed now carries a registered geographical indication.^8^ Food-related research on the crop nevertheless remains limited, largely because cultivation is restricted in many countries by its association with opium.^9,10^

Seed coat color is the trait by which poppy is graded and traded. It is primarily genotype-controlled: dark (gray to blue-black) cultivars accumulate flavonoid-derived pigments, mainly anthocyanin-type compounds, in the seed coat, whereas white- and yellow-seeded cultivars contain these pigments only in trace amounts.^11,12^ Growing conditions modulate the final hue, but the color class itself is genetically fixed, and commercial lots are classified and traded accordingly.^11,13^ Because the pigments concerned are phenolic, seed coat color is also expected to track the phenolic content and antioxidant potential of the seed and of the products derived from it.^12,14^

Cold pressing yields a high-value culinary oil and, alongside it, a substantial press cake. Oilseed cakes have historically been directed to animal feed, and poppy cake is recognized as a viable alternative feed protein source; although it holds clear promise for food fortification, descriptions of its composition and functional attributes remain fragmentary, with most assessments confined to a single species or a single matrix.^15,16^ Seed coat color in particular has rarely been treated as an experimental variable. Consequently, the compositional consequences of cold pressing cannot at present be separated from those of genotype, growing conditions, or analytical protocol.

Previous studies on poppy have addressed only the seed and/or the oil,^5,6,11,13,17–19^ characterized the press cake without matched seed and oil data,^15,16,20^ or characterized the seed, oil, and cake of a single variety without considering seed color.^4^ To the best of our knowledge, no study has yet characterized the whole seed, the cold-pressed oil, and the press-cake flour of white, yellow, and blue poppy seeds side by side, using the same pressing operation and the same analytical protocols, and evaluated the seed color × material interaction statistically. The present study was designed to fill this gap. The whole poppy seeds (WPSs), cold-pressed oils (CPOs), and press-cake flours (PSCFs) of three commercial color varieties (white, yellow, and blue) were analyzed in parallel for proximate composition, color, mineral profile, fatty acid composition, free amino acid profile, total phenolic content, and antioxidant capacity, together with the thermal and oxidative stability of the oils. The data were evaluated by two-way ANOVA so that each difference observed could be attributed to the pressing step, to seed coat color, or to their interaction. This study therefore contributes to the literature (i) a complete, matrix-matched compositional data set for poppy seed and its two cold-pressing products, (ii) the first free amino acid and mineral profiles of poppy press-cake flours differentiated by seed color, and (iii) quantitative evidence of how cold pressing redistributes nutrients and antioxidants between oil and cake, which is needed to guide the valorization of poppy press cake as a functional food ingredient.

## Materials and Methods

### Raw Materials

Seeds of opium poppy (*P. somniferum* L., *Papaveraceae*) of three commercially available color varieties (white, yellow, and blue) were obtained from a local supplier (Yörem Afyon) in Afyonkarahisar, Türkiye. In Turkish poppy production, seed lots are graded by seed coat color rather than as formally registered cultivars, so the samples studied here correspond to commercial color designations rather than to genetically defined cultivars.^7,11,13^ Because the seed lots were obtained from the commercial market, differences in genotype, cultivation conditions (location, season, and agronomic practice), and commercial lot may also have contributed to the differences observed among the color varieties; this limitation is considered in the interpretation of the results. Seeds were cold-pressed on a laboratory-scale screw press (Model GM 1000, General Equipment and Engineering, Ankara, Türkiye) fitted with a temperature-controlled barrel, at a barrel temperature of 40 °C, a screw speed of 19 rpm, and a total pressing time of 30 min, with the outlet oil temperature held below 50 °C. For each color, two separate seed lots (approximately 5 kg each) were processed as independent replicates: each lot was homogenized and cold-pressed in its own pressing run, so that the whole seed, the oil, and the press cake of a given replicate originate from the same batch of seed. Crude oil was separated from solids by sedimentation followed by filtration. The press cake was collected, ground through a 1 mm screen, and stored at 4 °C in airtight containers until analysis. All samples were subsampled from these batches for the analyses described below.

### Proximate Composition of Seeds and Press-Cake Flours

Moisture and ash contents were determined by AOAC^21^ methods 930.15 and 942.05, respectively; samples (2 g) were dried at 105 °C (Wiseven WON105) to constant mass. Crude protein content was determined by the Kjeldahl method according to the AACC method 46–10,^22^ using sulfuric acid digestion in the presence of catalyst tablets, alkaline distillation, and titration of the liberated ammonia, with a nitrogen-to-protein conversion factor of 6.25. Total lipid content was determined gravimetrically after Soxhlet extraction of approximately 5 g of sample with petroleum ether at 80 °C for 6–8 h. Color values (L*, a*, and b*) of seeds, oils, and cakes were measured with a Chroma Meter CR-400 (Konica Minolta Sensing, Osaka, Japan) in the CIELAB space,^23^ calibrated against a standard white plate; each sample was measured five times at different surface positions, and results are reported as mean ± SD L* is lightness (0 = black, 100 = white), a* the red (+)/green (−) axis, and b* the yellow (+)/blue (−) axis.

### Mineral Composition of Seeds and Press-Cake Flours

Mineral composition was determined as described by Adal et al.^24^ Moisture-free samples (0.2–0.3 g) were digested with nitric acid and hydrogen peroxide in Teflon vessels (Berghof Speedwave MWS-2) and the digests analyzed by inductively coupled plasma mass spectrometry (ICP-MS; Thermo Scientific iCAP-Q). Digestion was performed with HNO_3_ (65%, Suprapur) and H_2_O_2_ (30%) using a program consisting of a 15 min ramp to 180 °C, followed by a 20 min hold at 180 °C. The ICP-MS was operated in kinetic energy discrimination (KED) mode with helium as the collision gas to minimize polyatomic interferences. Values below the limit of detection are reported as not detected (N.D.). Each replicate digest was analyzed in duplicate. Digest concentrations (mg/L) were converted to sample concentrations (mg/kg) from the sample mass (0.25 g) and the final digest volume (25 mL). Minerals were determined in the solid fractions only because inorganic elements are largely bound to the protein–fiber matrix of the seed, are not coextracted with the lipid phase during cold pressing, and therefore occur in cold-pressed oils at nutritionally negligible trace levels; no mineral values are consequently reported for the CPOs.

### Fatty Acid Composition of Seeds, Oils, and Press Cakes

Fatty acid methyl esters (FAMEs) were prepared from the lipid fraction of the seeds, press cakes, and oils following the method proposed by Kivrak et al.^25^ and modified by Çetintaş et al.^26^ Briefly, 100 mg of oil (or of the petroleum-ether extract of the ground seeds and press cakes) was mixed with 10 mL of *n*-hexane and 100 μL of 2 N methanolic potassium hydroxide, vortexed, centrifuged, and filtered through a 0.20 μm syringe filter. FAMEs were analyzed on an Agilent 7890A gas chromatograph coupled to a 5975C inert mass selective detector (Agilent Technologies, Santa Clara, CA, USA) fitted with a DB-1 capillary column (30 m × 0.25 mm i.d., 0.25 μm film thickness; Agilent J&W). Helium was the carrier gas at 2.1 mL/min; 5 μL of sample was injected through a multimode inlet held at 280 °C with a split ratio of 40:1. The oven temperature was held at 110 °C for 1 min, raised at 3 °C/min to 290 °C, and held for 39 min. The mass spectrometer was operated in electron impact mode (70 eV) with the transfer line at 280 °C. Compounds were identified by comparing their retention times and mass spectra with those of the NIST and Wiley spectral libraries, and results are expressed as relative percentages of total fatty acids (FAs). The fatty acid profile was determined in all three fractions, rather than in the oil alone, because the lipid retained in press cake is not necessarily identical to the oil expressed from it; comparing the fractions therefore reveals whether triacylglycerol species partition selectively between oil and cake during pressing.

### Free Amino Acid Analysis

Free amino acids were quantified by liquid chromatography–tandem mass spectrometry (LC–MS/MS) following the method proposed by Çelik et al.^27^ and modified by Adal et al.^24^ Ground samples (200 mg) were mixed with 2 mL of ultrapure water acidified with 1% (v/v) formic acid, vortexed for 5 s, and sonicated for 10 min in an ultrasonic bath at 45 °C; the original method used the same aqueous extraction but a sonication time of 15 min at 45 °C. The extracts were centrifuged at 17,117*g* for 5 min (Nüve NF800R). A 50 μL portion of the clear supernatant was mixed with 50 μL of an internal standard solution containing deuterium-labeled amino acid analogues and 700 μL of a diluent composed of mobile phase A, methanol, and acetonitrile (5:15:15, v/v/v). The mixture was centrifuged again at 17,117 × g for 5 min, and the supernatant was transferred to glass autosampler vials for LC–MS/MS analysis.

Chromatographic separation was performed on an Agilent 1260 Infinity liquid chromatograph fitted with a Jasem amino acid column held at 30 °C, at a flow rate of 0.700 mL/min, and an injection volume of 5 μL. The gradient was 22% A/78% B at 1.0 min, 70% A/30% B at 4.0 min, held until 4.5 min, returned to 22% A/78% B at 4.6 min, and held until 7.5 min, giving a total run time of 9.0 min. Detection was performed on an Agilent 6460 triple-quadrupole mass spectrometer equipped with an Agilent Jet Stream electrospray ionization (AJS-ESI) source operated in positive-ion multiple reaction monitoring (MRM) mode. The parameters for the source were the following: drying-gas temperature at 150 °C; drying-gas flow rate of 10 L/min; nebulizer pressure set to 40 psi; sheath-gas temperature at 375 °C; sheath-gas flow rate of 10 L/min; and capillary voltage maintained at 2500 V. Compound-specific precursor/product-ion transitions, fragmentor voltages, and collision energies were used for MRM detection. Analytes were identified by matching their retention times and MRM transitions with those of reference standards and were quantified against internal-standard-corrected calibration curves. Data were acquired and processed with MassHunter software (Agilent Technologies). The values reported, therefore, represent the free amino acid pool and not the total (protein-bound plus free) amino acid composition.

### Total Phenolic Content

Extracts were prepared as described by Levent et al.:^28^ 1 g of ground seed, press-cake flour, or oil was mixed with 10 mL of 80% aqueous methanol (1:10, w/v), held at room temperature for 2 h, and centrifuged at 2600*g* for 15 min. The same extracts were used for the TPC and the three antioxidant capacity assays, all of which were read on a Multiskan GO microplate reader (Thermo Fisher Scientific, Vantaa, Finland). Total phenolic content (TPC) was determined by the Folin–Ciocalteu colorimetric assay as described by Slinkard and Singleton^29^ and Singleton and Rossi^30^ using gallic acid (0.1–8.0 mg/mL in methanol) as the standard. Results are expressed as gallic acid equivalents per milliliter of extract (mg GAE/mL).

### Antioxidant Activity Analyses

#### DPPH Radical-Scavenging Assay

Antioxidant activity was measured by the DPPH (1,1-diphenyl-2-picrylhydrazyl) radical-scavenging assay as described by Benvenuti et al.,^31^ with Trolox (6-hydroxy-2,5,7,8-tetramethylchroman-2-carboxylic acid; 0.1–4.0 mg/mL in ethanol) as the reference standard. Results are expressed as Trolox equivalents per milliliter of extract (mg TE/mL) and, additionally, as percentage inhibition of the radical.

#### FRAP Assay

The ferric reducing antioxidant power (FRAP) assay was performed according to the method proposed by Benzie and Strain^32^ and adapted by Benzie and Szeto,^33^ with Trolox (0.2–4.0 mg/mL in ethanol) as the standard. Results are expressed as mg TE/mL.

#### CUPRAC Assay

The cupric reducing antioxidant capacity (CUPRAC) assay, based on the reduction of Cu^2+^ to Cu^+^ by antioxidants in the presence of neocuproine, was carried out as a modification of the procedure of Apak et al.^34^ adapted to the microplate format. In each well, 50 μL of copper­(II) chloride solution (0.02 M in distilled water), 50 μL of neocuproine solution (0.015 M in ethanol), and 50 μL of ammonium acetate buffer (2 M in distilled water, pH 6.5) were mixed with 40 μL of extract (or Trolox standard) and 10 μL of distilled water to a final volume of 200 μL. The mixture was incubated for 30 min at room temperature in the dark, and the absorbance was read at 450 nm against a reagent blank. Trolox in ethanol was used as the calibration standard, and results are expressed as mg TE/mL.

#### Oil Quality and Stability Analyses

Refractive index, color, and viscosity of the oils were determined as described by Adal^23^ using an Abbe refractometer (RX-5000α, Atago Co., Ltd., Tokyo, Japan) at 20 °C, a Chroma Meter CR-400 (Konica Minolta Sensing, Osaka, Japan), and a vibro viscometer (SV-10, A&D Company, Ltd., Tokyo, Japan) at 25 °C, respectively. Thermal behavior was assessed by differential scanning calorimetry (DSC) following the method proposed by Tan and Che Man^35,36^ and modified by Adal.^23^ A DSC 8000 calorimeter (PerkinElmer, Waltham, MA, USA) calibrated with indium was used; oil samples were weighed into aluminum pans and hermetically sealed, an empty sealed pan served as a reference, and dry nitrogen (50 mL/min) was used as the purge gas. The onset, peak, and endset temperatures and the associated enthalpy changes (Δ*H*, J/g) of each transition were obtained from the thermograms with the instrument software. Oxidative stability was determined following the method proposed by Şahin et al.^37^ and modified by Adal,^23^ on a Rancimat 743 apparatus (Metrohm AG, Herisau, Switzerland). Oil samples (3.0 ± 0.1 g) were heated at 120 ± 0.1 °C under an air flow of 20 L/h, and the induction time (h) was recorded automatically from the inflection point of the conductivity curve.^38^

#### Experimental Design and Statistical Analysis

The study followed a 3 × 3 factorial design in which factor 1 was seed color (white, yellow, and blue) and factor 2 was material (whole seed, press-cake flour, and cold-pressed oil). Two independent replicates were conducted for each seed color. For each replicate, a separate seed lot was independently homogenized and cold-pressed, as described under Raw Materials, so that the seed, flour, and oil fractions within each replicate originated from the same batch. Thus, the two replicates represented independent experimental replicates rather than subsamples from a single pressing run. All determinations were carried out on subsamples of the corresponding fraction of each replicate; for color analysis, five readings were taken at different positions per sample and averaged within each replicate. Results are therefore expressed as the mean of the two independent replicates ± standard deviation (SD) (*n* = 2). The effects of seed color (C), material (M), and their interaction (C × M) were evaluated by two-way analysis of variance (ANOVA; Type III sums of squares) using SPSS 20 (IBM Corp., Armonk, NY, USA). The significance of the interaction term was assessed first. Because the interaction was significant for almost all variables and the variances differed markedly among materials, simple effects were then examined separately: differences among seed colors within each material, and differences among materials within each seed color, were tested by one-way ANOVA followed by Tukey’s HSD test, and the statistical letters in the tables were assigned accordingly. Refractive index, viscosity, and oxidative stability, which were measured only in the oils, were analyzed by one-way ANOVA, followed by the Tukey’s HSD test. Nondetected values were treated as zero, and *p*-values below 0.05 were considered statistically significant.

## Results and Discussion

### Statistical Overview

Because the three seed-color lots (white, yellow, and blue) and the three fractions (whole seed, press-cake flour, and cold-pressed oil) were processed and analyzed, the data were evaluated by two-way ANOVA with seed color (C), material (M), and their interaction (C × M) as factors, and the resulting *p*-values are reported alongside the means in Tables 1–5. The material effect was significant (*p* < 0.001) for every variable measured in more than one fraction, confirming that cold pressing redistributes the composition of the seed in a systematic way. The color effect and the C × M interaction were also significant for almost all variables; the only exceptions were moisture (C, *p* = 0.099; C × M, *p* = 0.055) and the total unsaturated fatty acid fraction (C, *p* = 0.260). A significant interaction means that the magnitude, and in several cases, the direction, of the color differences depended on the fraction examined, and the comparisons in the following sections were therefore made within each material (lowercase letters in the tables) and within each seed color (uppercase letters) rather than on the marginal means.

### Proximate Composition and Color Characteristics of Seeds, Press-Cake Flours, and Oils

Moisture content was governed by the material effect alone (Table 1). The press-cake flours (PSCFs) contained 7.25–7.45% moisture, and the whole poppy seeds (WPSs) 5.06–5.95%, every flour being significantly moister than the seed from which it was derived (*p* < 0.05), whereas seed color had no significant influence within either fraction. The seed values are in line with the mean of 6.4% reported by Azcan et al.^17^ and below the 9.97–11.11% found by Ghafoor et al.,^6^ while the flour values exceed the 4.45% reported for a poppy seed cake by Kukade et al.^39^ The higher moisture of the PSCFs is attributable to postpressing handling and to the hygroscopic character of the partially defatted, protein- and fiber-rich cake matrix, which retains water more effectively than the oil-rich intact seed.^4^ Ash, fat, and protein contents, by contrast, responded to seed color, material, and their interaction (*p* < 0.001 in every case). Ash content was highest in BF (13.46%), followed by YF (13.34%), and WF (12.38%), all three differing significantly, and the same 1.5–1.9-fold enrichment relative to the seeds was observed for each color (BS 8.87% > WS 7.29% > YS 7.07%). Because cold pressing removes the oil fraction without introducing mineral matter, inorganic constituents are proportionally concentrated in the residual cake; the values obtained agree with the approximately 10% ash reported for poppy cake by Melo et al.,^4^ the 8.70–11.14% found in the defatted meals of three Turkish varieties by Yılmaz and Emir,^20^ and the 11.19% reported by Bárta et al.,^16^ for whom poppy ranked highest among eight oilseed-cake flours. Consistent with the observation that high-ash flours tend to be darker,^16^ BF was both the most mineral-rich and by far the darkest flour.

**Table 1:** Proximate and Mineral Composition of Poppy Seed Press-Cake Flours and Whole Poppy Seeds^a^,^b^

							*p* **-value (two-way ANOVA)**		
**parameter**	**WF**	**YF**	**BF**	**WS**	**YS**	**BS**	**C**	**M**	**C × M**
moisture content (%)	7.45^aA^ ± 0.30	7.25^aA^ ± 0.19	7.36^aA^ ± 0.06	5.06^aB^ ± 0.40	5.48^aB^ ± 0.01	5.95^aB^ ± 0.00	0.099	<0.001	0.055
ash content (%)	12.38^cA^ ± 0.01	13.34^bA^ ± 0.02	13.46^aA^ ± 0.01	7.29^bB^ ± 0.01	7.07^cB^ ± 0.01	8.87^aB^ ± 0.02	<0.001	<0.001	<0.001
fat content (%)	10.59^aB^ ± 0.02	9.72^cB^ ± 0.04	9.86^bB^ ± 0.00	32.49^aA^ ± 0.01	30.38^bA^ ± 0.01	26.71^cA^ ± 0.01	<0.001	<0.001	<0.001
protein content (%)	37.27^aA^ ± 0.08	37.45^aA^ ± 0.02	33.70^bA^ ± 0.01	20.77^bB^ ± 0.01	24.22^aB^ ± 0.01	19.58^bB^ ± 0.61	<0.001	<0.001	<0.001
Mg (mg/kg)	9580^aA^ ± 45	8998^bA^ ± 32	8187^cA^ ± 81	5207^aB^ ± 37	4521^bB^ ± 45	4201^cB^ ± 20	<0.001	<0.001	<0.001
P (mg/kg)	14076^aA^ ± 65	13173^bA^ ± 53	12272^cA^ ± 47	8085^aB^ ± 38	7273^bB^ ± 40	6518^cB^ ± 32	<0.001	<0.001	0.032
K (mg/kg)	11905^aA^ ± 53	10899^bA^ ± 23	10718^bA^ ± 48	6687^aB^ ± 20	4216^bB^ ± 32	4361^bB^ ± 59	<0.001	<0.001	<0.001
Ca (mg/kg)	14658^aA^ ± 62	13893^bA^ ± 27	14627^aA^ ± 95	7883^aB^ ± 40	7284^cB^ ± 30	7714^bB^ ± 40	<0.001	<0.001	0.021
Mn (mg/kg)	42.5^aA^ ± 0.7	32.0^bA^ ± 0.0	30.0^cA^ ± 0.0	0.5^aB^ ± 0.7	N.D.	N.D.	<0.001	<0.001	<0.001
Fe (mg/kg)	383^bA^ ± 4	373^bA^ ± 3	411^aA^ ± 1	307^aB^ ± 8	192^cB^ ± 4	237^bB^ ± 3	<0.001	<0.001	<0.001
Zn (mg/kg)	26.5^bA^ ± 2.1	44.5^aA^ ± 0.7	17.0^cA^ ± 0.0	N.D.	N.D.	N.D.	<0.001	<0.001	<0.001
Sr (mg/kg)	17.5^aA^ ± 4.9	3.5^bA^ ± 0.7	N.D.	N.D.	N.D.	N.D.	0.002	0.001	0.002
Al (mg/kg)	2544^bA^ ± 41	4680^aA^ ± 31	631^cB^ ± 13	910^bB^ ± 16	N.D.	11890^aA^ ± 38	<0.001	<0.001	<0.001

a Values are the mean of two independent replicates (*n* = 2) ± SD. Moisture content is expressed on a wet basis; ash, fat, and protein contents are expressed on a dry weight basis (% dw); minerals are expressed as mg/kg on a dry weight basis. Within a row, means bearing different lowercase superscript letters differ significantly among seed colors within the same material (WF, YF, BF; WS, YS, and BS), and means bearing different uppercase superscript letters differ significantly among materials within the same seed color (WF vs WS; YF vs YS; BF vs BS), as determined by one-way ANOVA within each material or seed color, respectively, followed by Tukey’s HSD test (*p* < 0.05). C, M, and C × M, *p*-values of the seed color main effect, the material main effect, and their interaction obtained by two-way ANOVA (Type III sums of squares); *p* < 0.05 was considered significant. N.D., not detected (treated as zero in the statistical analysis).

b Abbreviations: WF, white poppy seed press-cake flour; YF, yellow poppy seed press-cake flour; BF, blue poppy seed press-cake flour; WS, white poppy seed; YS, yellow poppy seed; BS, blue poppy seed.

Fat content differed markedly between the WPSs and the PSCFs, as expected from the oil-extraction step: the fat content of the flours was roughly one-third of that of the seeds, which by mass balance corresponds to the recovery of about 70–75% of the seed oil during pressing. The seeds decreased in the order WS (32.49%) > YS (30.38%) > BS (26.71%), all differing significantly (*p* < 0.05), whereas the flours retained 9.72–10.59% fat, WF being highest and YF lowest. Reported poppy seed oil contents span 33.9–48.1%, with pressure extraction yielding 16.9–31%,^40^ and other studies give 24.20–45.52%;^5,6,41^ the present seeds therefore lie at the lower end of the published range, and the residual 9.7–10.6% fat of the PSCFs is characteristic of cold-pressed cake, in which mechanical pressing leaves part of the oil in the meal.^4,16^ Protein content was 1.5–1.8 times higher in the flours than in the corresponding seeds: WF (37.27%) and YF (37.45%) were statistically equivalent, and BF was lower (33.70%), while among the seeds, YS (24.22%) exceeded WS (20.77%) and BS (19.58%), which did not differ from one another. The seed values agree with published figures of 19.3–22.7%,^2,17^ and the seed-to-cake increase reflects the concentration effect described by Melo et al.^4^ and Bárta et al.,^16^ removal of the lipid fraction raising protein and ash in proportion and leaving a protein- and mineral-rich ingredient. Notably, the flour ranking (WF ≈ YF > BF) did not simply reproduce the seed ranking (YS > WS ≈ BS). This is the pattern captured by the significant C × M interaction, and it suggests that the pressing efficiency, and hence the degree of concentration, differed slightly among the color lots. Lot-to-lot differences in genotype and cultivation conditions may also have contributed to this pattern, since commercial color-graded seed lots were used.

Color values were affected by seed color, material, and their interaction for all three CIELAB coordinates (*p* < 0.001; Table 2). Lightness (L*) ranged from 33.02 (BS) to 71.59 (WF). Among the flours, WF and YF were equally light (71.59 and 68.74) and BF markedly darker (39.09); among the seeds, WS (60.78) was lighter than YS (41.23), which in turn was lighter than BS (33.02); and among the oils, YO (60.63) was lighter than WO (50.05), with BO intermediate (55.48). The interaction is evident in the direction of the material effect: for the white and yellow lots, the flour was the lightest fraction and the oil or seed the darkest, whereas for the blue lot, the oil was significantly lighter than both the seed and the flour, showing that the dark seed coat pigments remained almost entirely in the solid fractions during pressing. Redness (a*) was statistically uniform across the three flours (6.38–6.97) and, among the seeds, highest in YS (6.25) and lowest in BS (1.27); the oils were far less red (0.51–2.54), BO being the reddest and WO the least red. Yellowness (b*) spanned 1.91–17.12. WF and YF (16.63–16.95) and WS (17.12) were the most yellow samples, YS (14.98) being slightly but significantly less yellow than WS, and all four differed markedly from BF (5.82) and BS (1.91); among the oils, WO and YO (7.33–8.46) were more yellow than BO (2.45). Therefore, the white and yellow flours were lighter and yellower than the blue one, and the same held for the seeds and oils. This may be due to the influence of seed color on the hue of the obtained flour and oil.^13^ On the other hand, the residual oil brightens the color of the flour.^13^ The types and concentration of pigments in the blue-gray seed coat contribute to the darker and less yellow color of the products obtained from blue poppy seeds.^4^ The association between high ash content and dark color noted by Bárta et al.^16^ is evident here, and the variety-dependent seed coloration agrees with Apaydin.^2^ Color values reported for poppy seed oil by Martínez et al.^41^ (L* = 92.87, a* = – 4.66, b* = 23.69) are far removed from those measured here (L* = 50.05–60.63; a* = 0.51–2.54; b* = 2.45–8.46). The discrepancy is most plausibly methodological. Clear oils are usually measured in transmittance through a cuvette on fully clarified material, which yields high lightness and green–yellow tones from dissolved carotenoid and chlorophyll pigments. In contrast, surface-reflectance measurement, or measurement on unfiltered turbid oil in which suspended particles scatter light, gives the lower lightness and subdued hue obtained here.^42^ Cultivar, seed color, growing conditions, and pressing temperature modulate the result further.^4,13^

**Table 2:** Color Characteristics of Poppy Seeds, Poppy Seed Press-Cake Flours, and Cold-Pressed Poppy Seed Oils^a^,^b^

**sample**	***L*** *	***a*** *	***b*** *
WF	71.59^aA^ ± 0.80	6.63^aA^ ± 0.08	16.95^aA^ ± 0.13
YF	68.74^aA^ ± 0.07	6.97^aA^ ± 0.18	16.63^aA^ ± 0.50
BF	39.09^bB^ ± 2.74	6.38^aA^ ± 0.81	5.82^bA^ ± 1.14
WS	60.78^aB^ ± 0.37	5.02^bB^ ± 0.08	17.12^aA^ ± 0.17
YS	41.23^bC^ ± 0.25	6.25^aA^ ± 0.35	14.98^bA^ ± 0.46
BS	33.02^cB^ ± 1.40	1.27^cB^ ± 0.05	1.91^cB^ ± 0.03
WO	50.05^bC^ ± 1.52	0.51^cC^ ± 0.08	8.46^aB^ ± 0.76
YO	60.63^aB^ ± 2.76	1.38^bB^ ± 0.01	7.33^aB^ ± 0.64
BO	55.48^abA^ ± 0.66	2.54^aB^ ± 0.08	2.45^bB^ ± 0.28
*p* (C)	<0.001	<0.001	<0.001
*p* (M)	<0.001	<0.001	<0.001
*p* (C × M)	<0.001	<0.001	<0.001

a Values are the mean of two independent replicates (*n* = 2), each the average of five readings, ±SD. Within a column, means bearing different lowercase superscript letters differ significantly among seed colors within the same material (WF, YF, BF; WS, YS, BS; WO, YO, BO), and means bearing different uppercase superscript letters differ significantly among materials within the same seed color (WF, WS, WO; YF, YS, YO; BF, BS, BO), as determined by one-way ANOVA within each material or seed color, respectively, followed by Tukey’s HSD test (*p* < 0.05). C, M, and C × M, *p*-values of the seed color main effect, the material main effect, and their interaction obtained by two-way ANOVA (Type III sums of squares); *p* < 0.05 was considered significant. N.D., not detected (treated as zero in the statistical analysis).

b Abbreviations: WF, white poppy seed press-cake flour; YF, yellow poppy seed press-cake flour; BF, blue poppy seed press-cake flour; WS, white poppy seed; YS, yellow poppy seed; BS, blue poppy seed; WO, white poppy seed cold-pressed oil; YO, yellow poppy seed cold-pressed oil; and BO, blue poppy seed cold-pressed oil.

### Mineral Content of Seeds and Press-Cake Flours

Minerals were determined in the two solid fractions only, for the reasons given in Materials and Methods, and are reported as mg/kg (Table 1). Seed color, material, and their interaction were significant for all nine elements (*p* ≤ 0.032), and for every element except aluminum, the flour contained a significantly higher concentration than the corresponding seed, so that the byproduct of oil extraction acts as a concentrated mineral source. The same effect has been described for poppy press cake by Yılmaz and Emir,^20^ who attributed the mineral load of defatted poppy meals chiefly to K, Na, Ca, Mg, and Zn, and by Melo et al.,^4^ who found that partial defatting raises the relative concentration of all nonlipid components and lifts cake ash to around 10%.

The four macroelements followed a consistent flour > seed pattern, the enrichment factor being 1.7–2.0 for Mg, P, and Ca and 1.8–2.6 for K. Magnesium, phosphorus, and potassium were all highest in WF (9580, 14,076, and 11,905 mg/kg, respectively) and decreased in the order WF > YF > BF for Mg and P and WF > YF ≈ BF for K. Calcium was highest and statistically equivalent in WF and BF (14,658 and 14,627 mg/kg) and lower in YF (13,893 mg/kg). The seeds showed the same color ranking, WS being richest in all four elements (5207 mg/kg Mg, 8085 mg/kg P, 6687 mg/kg K, and 7883 mg/kg Ca) and BS or YS poorest (4201 mg/kg Mg and 6518 mg/kg P in BS; 4216 mg/kg K and 7284 mg/kg Ca in YS). These four elements consistently dominate the poppy mineral profile irrespective of cultivar: Melo et al.^4^ compiled ranges of approximately 8757–10,702 mg/kg Ca, 9081–10,536 mg/kg P, 6012–10,536 mg/kg K, and 3407–3872 mg/kg Mg for poppy seed, and comparable values have been reported by Harmankaya et al.^43^ and Özcan.^44^ The present seed values fall within or slightly above these ranges, and the flour values exceed them in proportion to the oil removed. The predominance of these four elements is nutritionally relevant. Calcium and phosphorus are the principal mineral constituents of bone and teeth, and phosphorus is additionally required for nucleic acids, phospholipids and energy metabolism (ATP);^45^ magnesium acts as a cofactor of more than 300 enzymes, participates in neuromuscular function and glycaemic control, and is frequently under-consumed in Western diets;^46^ potassium, the main intracellular cation, is essential for fluid and electrolyte balance and for blood-pressure regulation, a high potassium intake being associated with a lower risk of hypertension and stroke.^47^ On the basis of the present data, 10 g of WF would supply roughly 147 mg Ca, 96 mg Mg, 141 mg P, and 119 mg K, i.e., about 15, 25, 20, and 3–4% of typical adult daily requirements, respectively, which underlines the potential of the flours as mineral-dense ingredients.

Among the microelements, manganese was essentially confined to the flours (WF 42.5 > YF 32.0 > BF 30.0 mg/kg), the seeds containing at most 0.5 mg/kg. Iron was highest in BF (411 mg/kg), which exceeded WF and YF (383 and 373 mg/kg, statistically equivalent), whereas among the seeds, WS (307 mg/kg) was richest and YS (192 mg/kg) poorest. Zinc was detected only in the flours, in the order YF (44.5) > WF (26.5) > BF (17.0 mg/kg), and strontium only in WF (17.5 mg/kg) and YF (3.5 mg/kg). Among these microelements, iron is required for hemoglobin and myoglobin synthesis and oxygen transport, and iron deficiency remains the most widespread nutritional deficiency worldwide;^48^ zinc is a structural and catalytic component of several hundred enzymes and transcription factors and is essential for growth, immune function, and wound healing;^49^ and manganese is a cofactor of mitochondrial superoxide dismutase, arginase, and pyruvate carboxylase and thus participates in antioxidant defense, bone formation, and carbohydrate metabolism.^50^ The iron content of the flours (373–411 mg/kg) is particularly noteworthy, as 10 g of flour would provide about 4 mg of Fe, roughly half of the adult male requirement, although the bioavailability of nonheme iron from a phytate-rich matrix such as oilseed cake is expected to be limited. Aluminum followed a distinct pattern, the highest concentration by far being recorded in BS (11,890 mg/kg), which was 13 times higher than in WS (910 mg/kg) and 19 times higher than in the flour derived from it (BF, 631 mg/kg), while YS contained no detectable Al and among the flours YF (4680 mg/kg) exceeded WF (2544 mg/kg). Aluminum is not an essential element,^51^ and concentrations of this order in seeds are more commonly associated with adhering soil or dust particles, or with contact surfaces during harvesting and processing, than with physiological uptake;^52,53^ the very different Al levels of the seed and flour of the same color lot support an external origin and indicate that this parameter should be verified in independent lots before any nutritional conclusion is drawn.

The varietal differences observed, the Zn and Sr enrichment of the yellow and white flours, the Fe enrichment of BF, and the genotype-dependent macroelement levels, are consistent with evidence that poppy seed composition is governed largely by genotype and growing conditions. Rahimi et al.^18^ showed that four registered Turkish cultivars differed significantly by genotype and location, and Adal et al.^24^ found Ca, Mg, and Fe to differ markedly among blue-, yellow-, and white-seeded poppy press-cake flours. Overall, the PSCFs were richer than the whole seeds in Mg, P, K, Ca, Mn, Fe, and Zn, with WF generally highest for the macroelements and YF for Zn, which supports their growing use as a fortifying ingredient: poppy seed cake has been shown to raise the protein, fiber, and mineral content of oat cookies without loss of sensory acceptability,^54^ to improve the nutritional quality of gluten-free baked goods,^55^ and to increase the calcium, magnesium, and iron content of gluten-free cookies.^24^

### Fatty Acid Composition of Seeds, Oils, and Press Cakes

Linoleic acid was the predominant fatty acid in every matrix (64.41–76.81% of total FAs) and oleic acid, consistently the second most abundant (12.33–15.80%), confirming that poppy-derived products are rich in polyunsaturated fatty acids (PUFAs; Table 3). The material effect was significant for every fatty acid (*p* < 0.001), and seed color and the C × M interaction were significant for all of them as well, although for linoleic acid, the color effect was comparatively weak (*p* = 0.018; C × M, *p* = 0.005). Linoleic acid was highest in YO (76.81%) and, among the seeds, in WS (71.07%), which exceeded BS and YS (66.01–66.51%); within the flours (64.41–66.20%) and within the oils (69.45–76.81%), the color lots did not differ significantly. The oils and seeds generally carried more linoleic acid than the flours, WS exceeded WF, YO exceeded YF and YS, and BO exceeded BF, indicating that the lipid remaining in the cake is proportionally poorer in this fatty acid. The values are consistent with the classification of poppy seed oil as a linoleic oil containing 69–77% C18:2, 13–18% C18:1, and 9–11% C16:0,^56^ with the 69.85–74.02% linoleic acid reported by Aksoylu Özbek and Ergönül^19^ for expeller-pressed oils of five Turkish varieties, and with the triacylglycerol profiles dominated by linoleic-acid-rich species that Krist et al.^57^ found in the seed oils of a range of poppy varieties. Oleic acid showed the opposite color preference: it was highest in the blue products of each fraction (BF 15.58%, BS 15.80%, and BO 15.32%, the latter two statistically equivalent to YS and YO, respectively) and lowest in YF (12.33%), and Bardeau et al.^58^ similarly found modest oleic acid in poppy press cake and oil (13.9 and 14.3%) against a clear predominance of linoleic acid.

**Table 3:** Fatty Acid (FA) Contents (% of Total FAs) and Calculated Nutritional Indices of Poppy Seeds, Poppy Seed Press-Cake Flours, and Cold-Pressed Poppy Seed Oils^a^,^b^,^c^

										*p* **-value (two-way ANOVA)**		
**common name**	**WF**	**YF**	**BF**	**WS**	**YS**	**BS**	**WO**	**YO**	**BO**	**C**	**M**	**C × M**
myristic acid (C14:0)	N.D.	N.D.	N.D.	0.06^aA^ ± 0.01	0.05^aA^ ± 0.00	0.07^aA^ ± 0.00	N.D.	N.D.	0.04^aB^ ± 0.00	<0.001	<0.001	<0.001
palmitoleic acid (C16:1 *n*-7)	0.13^cB^ ± 0.00	0.33^aA^ ± 0.01	0.17^bA^ ± 0.01	0.16^abA^ ± 0.00	0.16^bB^ ± 0.00	0.17^aA^ ± 0.00	0.14^aB^ ± 0.00	N.D.	0.12^bB^ ± 0.00	<0.001	<0.001	<0.001
palmitic acid (C16:0)	13.08^bA^ ± 0.06	13.75^aA^ ± 0.21	14.00^aA^ ± 0.11	10.25^cC^ ± 0.27	11.46^bB^ ± 0.17	12.75^aB^ ± 0.12	11.13^aB^ ± 0.03	8.70^cC^ ± 0.17	10.02^bC^ ± 0.22	<0.001	<0.001	<0.001
margaric acid (C17:0)	N.D.	0.11^aA^ ± 0.00	0.09^bA^ ± 0.00	0.05^bA^ ± 0.00	0.04^cB^ ± 0.00	0.07^aB^ ± 0.00	0.04^aB^ ± 0.00	N.D.	N.D.	<0.001	<0.001	<0.001
linoleic acid (C18:2 *n*-6)	64.41^aB^ ± 0.90	66.20^aB^ ± 1.92	64.70^aB^ ± 0.84	71.07^aA^ ± 0.30	66.51^bB^ ± 1.81	66.01^bAB^ ± 0.02	69.93^aAB^ ± 2.36	76.81^aA^ ± 2.18	69.45^aA^ ± 1.20	0.018	<0.001	0.005
oleic acid (C18:1 *n*-9)	14.37^bA^ ± 0.16	12.33^cC^ ± 0.17	15.58^aA^ ± 0.02	13.68^bAB^ ± 0.05	15.73^aA^ ± 0.36	15.80^aA^ ± 0.51	13.38^bB^ ± 0.26	14.13^abB^ ± 0.43	15.32^aA^ ± 0.42	<0.001	<0.001	<0.001
elaidic acid (C18:1 trans *n*-9)	1.62^aA^ ± 0.01	1.64^aA^ ± 0.05	1.67^aA^ ± 0.05	1.32^aB^ ± 0.03	1.34^aB^ ± 0.04	1.44^aB^ ± 0.02	1.24^aC^ ± 0.01	0.70^bC^ ± 0.01	1.26^aC^ ± 0.03	<0.001	<0.001	<0.001
stearic acid (C18:0)	5.79^aA^ ± 0.06	4.01^bA^ ± 0.06	3.87^bA^ ± 0.01	2.76^bB^ ± 0.07	2.90^bB^ ± 0.06	3.48^aB^ ± 0.11	2.57^bB^ ± 0.09	1.65^cC^ ± 0.03	2.89^aC^ ± 0.06	<0.001	<0.001	<0.001
gondoic acid (C20:1 *n*-9)	N.D.	N.D.	0.17^aA^ ± 0.00	0.09^bA^ ± 0.00	0.07^cA^ ± 0.00	0.14^aB^ ± 0.00	N.D.	N.D.	N.D.	<0.001	<0.001	<0.001
arachidic acid (C20:0)	N.D.	N.D.	0.27^aA^ ± 0.01	0.13^bA^ ± 0.00	0.11^cA^ ± 0.00	0.20^aB^ ± 0.00	0.12^bB^ ± 0.00	N.D.	0.13^aC^ ± 0.00	<0.001	<0.001	<0.001
behenic acid (C22:0)	N.D.	N.D.	0.10^aA^ ± 0.00	N.D.	N.D.	0.03^aB^ ± 0.00	N.D.	N.D.	N.D.	<0.001	<0.001	<0.001
lignoceric acid (C24:0)	N.D.	N.D.	0.05^aA^ ± 0.00	N.D.	N.D.	0.02^aB^ ± 0.00	N.D.	N.D.	N.D.	<0.001	<0.001	<0.001
∑SFAs	18.88^aA^ ± 0.12	17.86^bA^ ± 0.27	18.38^abA^ ± 0.11	13.24^cB^ ± 0.33	14.56^bB^ ± 0.11	16.61^aB^ ± 0.22	13.86^aB^ ± 0.05	10.34^bC^ ± 0.20	13.07^aC^ ± 0.27	<0.001	<0.001	<0.001
∑MUFAs	16.12^bA^ ± 0.18	14.30^cB^ ± 0.13	17.58^aA^ ± 0.02	15.26^bB^ ± 0.02	17.29^aA^ ± 0.40	17.55^aA^ ± 0.49	14.75^bB^ ± 0.24	14.83^bB^ ± 0.42	16.70^aA^ ± 0.45	<0.001	<0.001	<0.001
∑PUFAs	64.41^aB^ ± 0.90	66.20^aB^ ± 1.92	64.70^aB^ ± 0.84	71.07^aA^ ± 0.30	66.51^bB^ ± 1.81	66.01^bAB^ ± 0.02	69.93^aAB^ ± 2.36	76.81^aA^ ± 2.18	69.45^aA^ ± 1.20	0.018	<0.001	0.005
∑UFAs	80.53^aB^ ± 0.72	80.50^aB^ ± 2.05	82.28^aB^ ± 0.82	86.32^aA^ ± 0.28	83.80^aAB^ ± 2.21	83.56^aAB^ ± 0.46	84.68^aAB^ ± 2.11	91.64^aA^ ± 2.60	86.16^aA^ ± 0.75	0.260	<0.001	0.012
UFAs/SFAs	4.27^bB^ ± 0.01	4.51^aC^ ± 0.05	4.48^aC^ ± 0.02	6.52^aA^ ± 0.14	5.76^bB^ ± 0.11	5.03^cB^ ± 0.10	6.11^bA^ ± 0.18	8.86^aA^ ± 0.08	6.59^bA^ ± 0.08	<0.001	<0.001	<0.001
PUFAs/SFAs	3.41^bB^ ± 0.03	3.71^aC^ ± 0.05	3.52^bC^ ± 0.02	5.37^aA^ ± 0.11	4.57^bB^ ± 0.09	3.98^cB^ ± 0.05	5.05^bA^ ± 0.19	7.43^aA^ ± 0.06	5.31^bA^ ± 0.02	<0.001	<0.001	<0.001
ω-3/ω-6	0.00	0.00	0.00	0.00	0.00	0.00	0.00	0.00	0.00			

a Values are the mean of two independent replicates (*n* = 2) ± SD. Within a row, means bearing different lowercase superscript letters differ significantly among seed colors within the same material (WF, YF, BF; WS, YS, BS; WO, YO, and BO), and means bearing different uppercase superscript letters differ significantly among materials within the same seed color (WF, WS, WO; YF, YS, YO; BF, BS, and BO), as determined by one-way ANOVA within each material or seed color, respectively, followed by Tukey’s HSD test (*p* < 0.05). C, M, and C × M, *p*-values of the seed color main effect, the material main effect, and their interaction obtained by two-way ANOVA (Type III sums of squares); *p* < 0.05 was considered significant. N.D., not detected (treated as zero in the statistical analysis).

b Abbreviations: WF, white poppy seed press-cake flour; YF, yellow poppy seed press-cake flour; BF, blue poppy seed press-cake flour; WS, white poppy seed; YS, yellow poppy seed; BS, blue poppy seed; WO, white poppy seed cold-pressed oil; YO, yellow poppy seed cold-pressed oil; and BO, blue poppy seed cold-pressed oil.

c ∑SFAs, ∑MUFAs, ∑PUFAs, and ∑UFAs were calculated for each replicate as the sums of the individual saturated, monounsaturated, polyunsaturated, and unsaturated fatty acids listed above; the UFAs/SFAs and PUFAs/SFAs ratios were calculated from these sums, and the statistical analysis was applied to the replicate values. No ω-3 fatty acids were detected in any sample, so the ω-3/ω-6 ratio is zero throughout and was not analyzed statistically.

Among the saturated fatty acids (SFAs), palmitic acid was the most abundant and was significantly concentrated in the PSCFs (13.08–14.00%, BF and YF exceeding WF), whereas the seeds contained 10.25–12.75% (BS > YS > WS) and the oils 8.70–11.13% (WO > BO > YO). Stearic acid was likewise higher in the flours (3.87–5.79%, WF far exceeding YF and BF) than in the seeds (2.76–3.48%) and oils (1.65–2.89%), YO again being lowest. These findings agree with Azcan et al.,^17^ who reported markedly lower stearic (2.5–3.2%) and linolenic (0.4–0.6%) acids than palmitic (10.0–13.0%), oleic (16.1–24.7%), and linoleic (56.4–69.2%) acids in poppy seeds. The consistent enrichment of palmitic and stearic acid in the cake relative to both the seed and the expressed oil suggests that the more fluid, unsaturated triacylglycerols are expressed preferentially during cold pressing, leaving a residual cake lipid that is slightly more saturated than the parent seed oil. The minor fatty acids were strongly genotype-dependent. Myristic acid was detected only in the seeds (0.05–0.07%) and in BO (0.04%), palmitoleic acid was highest in YF (0.33%) and absent from YO, and margaric acid ranged from not detected (WF, YO, and BO) to 0.11% (YF). The very-long-chain saturated and monounsaturated acids, gondoic (C20:1), arachidic (C20:0), behenic (C22:0), and lignoceric (C24:0), were consistently highest in the blue lot: BF contained 0.17, 0.27, 0.10, and 0.05% and BS 0.14, 0.20, 0.03, and 0.02% of these acids, whereas behenic and lignoceric acids were absent from all white and yellow products. Variety-dependent minor lipid constituents of this kind have been reported previously for poppy seed oils of different varieties^11,57^ and may prove useful as compositional markers of the blue-seeded material. A small *trans*-C18:1 (elaidic acid) fraction was recorded in all samples (0.70–1.67%), highest in the flours and lowest in YO; since the seeds and oils were never exposed to temperatures above 50 °C, this signal is more likely to originate from coelution of positional C18:1 isomers under the chromatographic conditions used than from processing-induced isomerization, and it should be interpreted with caution.

The nutritional indices, calculated for each replicate and analyzed statistically (Table 3), placed ∑SFAs highest in the flours (17.86–18.88%), intermediate in the seeds (13.24–16.61%), and lowest in the oils (10.34–13.86%; WO and WS did not differ significantly), the yellow oil being the least saturated of all samples. ∑MUFAs ranged from 14.30% (YF) to 17.58% (BF), the blue products again being highest or joint-highest within each fraction. ∑UFAs varied between 80.50% (YF) and 91.64% (YO); the material effect and the interaction were significant (*p* < 0.001 and *p* = 0.012), but seed color as a main effect was not significant (*p* = 0.260), and within each fraction, the color lots did not differ significantly in ∑UFAs. WS showed the numerically highest proportion of UFAs among the seeds (86.32%), close to the 88.80–89.83% reported for poppy seed oil^59^ and the 87.59–89.78% range of Turkish opium poppy varieties.^60^ The UFAs/SFAs ratio ranged from 4.27 (WF) to 8.86 (YO), and the PUFAs/SFAs ratio from 3.41 (WF) to 7.43 (YO), the oils and seeds exceeding the flours in both indices; all values are well above the minimum PUFAs/SFAs ratio of 0.45 recommended for a healthy diet,^61^ confirming a favorable unsaturation profile across all matrices and particularly in the oils. No ω-3 fatty acids were detected in any sample, so the PUFA fraction consisted entirely of ω-6 linoleic acid, and the ω-3/ω-6 ratio was zero throughout, consistent with the very low linolenic acid contents (0.4–0.6%) previously reported for poppy seed.^17^ Poppy-derived products are therefore ω-6-dominant and fall below the ω-3/ω-6 ratio of ≥0.25 recommended for a balanced diet^62^ and are best regarded as a source of ω-6 PUFAs to be combined with ω-3-rich foods such as oily fish or flaxseed. Together with the residual oil and protein content of the cake (9.72–10.59% and 33.70–37.45%, respectively; Table 1), the favorable fatty acid profile supports the value of the press-cake fraction as a food and feed ingredient.

### Free Amino Acid Composition of Seeds and Press-Cake Flours

It should be emphasized that the data in Table 4 describe the free amino acid fraction, i.e., the amino acids extractable without prior hydrolysis, and not the total amino acid composition of the proteins; the two are not interchangeable and are discussed here strictly in terms of the free pool. The most striking feature of the data set is the size of the material effect: the total free amino acid pool amounted to 55,530–69,101 nmol/g in the PSCFs but only 570–1962 nmol/g in the WPSs, a 33 to 97-fold difference that far exceeds the 1.5- to 1.8-fold enrichment observed for protein and ash, i.e., the concentration attributable to removal of the oil fraction. Cold pressing therefore does more than concentrate the nonlipid matrix. The mechanical shear and the mild heating (barrel temperature 40 °C, oil outlet below 50 °C) associated with screw pressing evidently disrupt the seed cells. This either releases free amino acids from compartments that are inaccessible to direct aqueous extraction of the intact seed or promotes limited proteolysis of storage proteins. Whichever mechanism predominates, the free amino acid pool of the cake is a product of processing as much as of genotype, and this should be borne in mind when free amino acid data are used to compare seeds and cakes.

**Table 4:** Free Amino Acid Contents (nmol/g) of Poppy Seeds and Poppy Seed Press-Cake Flours^a^,^b^

							*p* **-value (two-way ANOVA)**		
**compound (nmol/g)**	**WF**	**YF**	**BF**	**WS**	**YS**	**BS**	**C**	**M**	**C × M**
L-tryptophan	330.6^aA^ ± 2.5	335.3^aA^ ± 2.8	235.2^bA^ ± 2.7	5.5^aB^ ± 0.1	1.6^bB^ ± 0.0	0.4^cB^ ± 0.0	<0.001	<0.001	<0.001
l-phenylalanine	1988.6^aA^ ± 5.3	1952.6^bA^ ± 4.6	1262.1^cA^ ± 5.2	60.7^aB^ ± 0.5	11.5^bB^ ± 0.1	3.8^cB^ ± 0.1	<0.001	<0.001	<0.001
l-tyrosine	767.8^bA^ ± 4.9	977.0^aA^ ± 6.2	651.1^cA^ ± 4.3	10.8^aB^ ± 0.1	6.2^bB^ ± 0.1	0.9^cB^ ± 0.0	<0.001	<0.001	<0.001
l-leucine	1499.2^bA^ ± 5.2	1666.7^aA^ ± 6.0	1178.5^cA^ ± 5.5	30.8^aB^ ± 0.2	13.2^bB^ ± 0.1	3.6^cB^ ± 0.0	<0.001	<0.001	<0.001
l-isoleucine	437.5^aA^ ± 2.8	434.9^aA^ ± 3.2	359.5^bA^ ± 2.2	14.9^aB^ ± 0.2	7.5^bB^ ± 0.0	1.4^cB^ ± 0.0	<0.001	<0.001	<0.001
l-methionine	602.8^bA^ ± 3.6	656.7^aA^ ± 3.7	423.2^cA^ ± 3.4	19.2^aB^ ± 0.1	4.2^bB^ ± 0.0	1.0^cB^ ± 0.0	<0.001	<0.001	<0.001
γ-aminobutyric acid	918.5^bA^ ± 6.1	1270.2^aA^ ± 6.7	837.9^cA^ ± 4.5	63.8^aB^ ± 0.5	42.8^bB^ ± 0.4	8.3^cB^ ± 0.1	<0.001	<0.001	<0.001
l-valine	1871.3^aA^ ± 6.1	1873.2^aA^ ± 6.0	1594.8^bA^ ± 5.2	31.9^aB^ ± 11	22.0^cB^ ± 0.2	26.9^bB^ ± 0.2	<0.001	<0.001	<0.001
l-2-aminoadipic acid	478.5^bA^ ± 3.4	350.7^cA^ ± 2.7	554.8^aA^ ± 3.8	10.6^aB^ ± 0.1	8.4^bB^ ± 0.1	2.4^cB^ ± 0.0	<0.001	<0.001	<0.001
l-glutamic acid	11293.0^bA^ ± 18.0	12336.5^aA^ ± 17.9	10860.6^cA^ ± 15.4	262.2^aB^ ± 1.0	144.2^cB^ ± 0.4	148.0^bB^ ± 0.4	<0.001	<0.001	<0.001
ethanolamine	729.0^aA^ ± 3.1	599.2^bA^ ± 2.7	432.5^cA^ ± 2.7	46.7^aB^ ± 0.3	25.7^bB^ ± 0.2	5.6^cB^ ± 0.1	<0.001	<0.001	<0.001
β-alanine	102.0^aA^ ± 0.4	89.6^bA^ ± 0.8	78.7^cA^ ± 0.5	13.2^aB^ ± 0.1	6.1^bB^ ± 0.0	2.0^cB^ ± 0.0	<0.001	<0.001	<0.001
l-aspartic acid	3188.2^cA^ ± 10.3	3814.3^aA^ ± 8.3	3423.0^bA^ ± 8.3	133.1^aB^ ± 0.6	71.6^bB^ ± 0.5	31.6^cB^ ± 0.3	<0.001	<0.001	<0.001
l-threonine	1362.6^bA^ ± 3.9	1430.4^aA^ ± 3.6	1268.3^cA^ ± 3.1	74.8^aB^ ± 0.2	52.0^bB^ ± 0.2	26.7^cB^ ± 0.1	<0.001	<0.001	<0.001
l-serine	1758.4^bA^ ± 5.3	1886.8^aA^ ± 5.3	1737.9^bA^ ± 5.5	81.5^aB^ ± 0.4	37.7^bB^ ± 0.3	23.8^cB^ ± 0.2	<0.001	<0.001	<0.001
l-alanine	6541.5^aA^ ± 12.5	6331.6^bA^ ± 12.0	5191.7^cA^ ± 9.8	224.7^aB^ ± 1.0	143.8^bB^ ± 0.6	41.1^cB^ ± 0.2	<0.001	<0.001	<0.001
l-glycine	2782.2^bA^ ± 9.6	3001.7^aA^ ± 9.4	2283.4^cA^ ± 5.9	100.3^bB^ ± 0.7	111.3^aB^ ± 0.6	60.5^cB^ ± 0.4	<0.001	<0.001	<0.001
trans-4-hydroxy-l-proline	1067.9^aA^ ± 4.3	917.8^bA^ ± 3.6	711.5^cA^ ± 3.0	42.0^aB^ ± 0.2	17.2^bB^ ± 0.1	5.6^cB^ ± 0.1	<0.001	<0.001	<0.001
l-asparagine	14222.8^aA^ ± 31.9	13812.6^bA^ ± 27.7	11004.9^cA^ ± 22.2	374.9^aB^ ± 1.3	182.8^bB^ ± 0.6	45.1^cB^ ± 0.2	<0.001	<0.001	<0.001
l-proline	3215.3^bA^ ± 7.9	3325.0^aA^ ± 7.9	3321.0^aA^ ± 7.0	57.9^aB^ ± 0.3	48.6^bB^ ± 0.2	10.7^cB^ ± 0.1	<0.001	<0.001	<0.001
l-glutamine	2253.5^bA^ ± 7.0	2488.0^aA^ ± 6.7	1719.6^cA^ ± 6.1	117.2^aB^ ± 0.3	94.0^bB^ ± 0.3	40.7^cB^ ± 0.2	<0.001	<0.001	<0.001
l-histidine	149.7^aA^ ± 0.8	139.7^bA^ ± 0.6	116.2^cA^ ± 0.5	33.9^aB^ ± 0.2	22.5^bB^ ± 0.1	12.6^cB^ ± 0.1	<0.001	<0.001	<0.001
l-arginine	5895.2^bA^ ± 10.1	7794.6^aA^ ± 9.6	5063.8^cA^ ± 7.8	86.3^bB^ ± 0.4	88.2^aB^ ± 0.3	42.7^cB^ ± 0.2	<0.001	<0.001	<0.001
argininosuccinic acid	30.3^aA^ ± 0.3	20.9^cA^ ± 0.2	23.9^bA^ ± 0.1	0.3^cB^ ± 0.0	2.4^aB^ ± 0.0	0.8^bB^ ± 0.0	<0.001	<0.001	<0.001
l-ornithine	45.8^aA^ ± 0.3	39.8^bA^ ± 0.4	31.7^cA^ ± 0.4	9.1^aB^ ± 0.1	6.5^bB^ ± 0.1	6.0^cB^ ± 0.0	<0.001	<0.001	<0.001
DL-5-hydroxylysine	7.9^cA^ ± 0.1	9.2^bA^ ± 0.1	14.9^aA^ ± 0.1	N.D.	N.D.	N.D.	<0.001	<0.001	<0.001
l-lysine	1284.5^bA^ ± 4.7	1521.7^aA^ ± 6.0	1132.9^cA^ ± 5.0	56.0^aB^ ± 0.2	30.7^bB^ ± 0.2	18.3^cB^ ± 0.1	<0.001	<0.001	<0.001
3-methyl-l-histidine	26.4^aA^ ± 0.2	24.8^bA^ ± 0.2	16.7^cA^ ± 0.1	N.D.	N.D.	N.D.	<0.001	<0.001	<0.001

a Values are the mean of two independent replicates (*n* = 2) ± SD and refer to the free amino acid fraction, extracted without acid hydrolysis; they are not the total (protein-bound plus free) amino acid composition. Within a row, means bearing different lowercase superscript letters differ significantly among seed colors within the same material (WF, YF, BF; WS, YS, and BS), and means bearing different uppercase superscript letters differ significantly among materials within the same seed color (WF vs WS; YF vs YS; BF vs BS), as determined by one-way ANOVA within each material or seed color, respectively, followed by Tukey’s HSD test (*p* < 0.05). C, M, and C × M, *p*-values of the seed color main effect, the material main effect, and their interaction obtained by two-way ANOVA (Type III sums of squares); *p* < 0.05 was considered significant. N.D., not detected (treated as zero in the statistical analysis).

b Abbreviations: WF, white poppy seed press-cake flour; YF, yellow poppy seed press-cake flour; BF, blue poppy seed press-cake flour; WS, white poppy seed; YS, yellow poppy seed; BS, blue poppy seed.

Within the flours, YF was the richest source overall (69,101 nmol/g), followed by WF (64,851 nmol/g) and BF (55,530 nmol/g), and the pool was dominated in all three by l-asparagine (approximately 20–22% of the total), l-glutamic acid (17–20%), l-alanine (9–10%), and l-arginine (9–11%). YF held the highest concentrations of the essential amino acids l-leucine, l-methionine, l-threonine, and l-lysine, of the conditionally essential l-tyrosine, and of γ-aminobutyric acid, l-glutamic acid, l-aspartic acid, l-serine, l-glycine, l-glutamine, and l-arginine (*p* < 0.05), and was statistically equivalent to WF for l-tryptophan, l-isoleucine, and l-valine. WF was richest in l-phenylalanine, l-alanine, ethanolamine, β-alanine, l-asparagine, trans-4-hydroxy-l-proline, l-histidine, argininosuccinic acid, l-ornithine, and 3-methyl-l-histidine, whereas BF, although lowest in most essential amino acids, carried the highest levels of l-2-aminoadipic acid (554.8 nmol/g) and DL-5-hydroxylysine (14.9 nmol/g). The essential amino acids accounted for 13.6–14.7% of the free pool in the flours, and the pattern of the essential fraction, high leucine, valine, and lysine but comparatively low methionine and tryptophan, mirrors the composition reported for the total protein of poppy seed, in which lysine has been identified as the limiting amino acid, and the chemical score was 60–66.^63^

In the whole seeds, the free amino acid levels were far lower, and seed color had a pronounced effect: WS contained the highest concentrations of almost every compound, led by l-asparagine (374.9 nmol/g), l-glutamic acid (262.2 nmol/g), and l-alanine (224.7 nmol/g), YS was intermediate, and BS was lowest for all but a few compounds, the exceptions being l-glycine, l-arginine, and argininosuccinic acid, for which YS was highest, and l-glutamic acid and l-valine, for which BS exceeded YS. DL-5-hydroxylysine and 3-methyl-l-histidine were not detected in any seed, although both were present in every flour, which again points to their release or formation during pressing. The seed ranking (WS > YS > BS) thus differed from the flour ranking (YF > WF > BF), the pattern captured by the significant C × M interaction. These observations are consistent with previous work on poppy seed cake. Tekeli et al.^64^ identified 17 amino acids, 10 of them essential, with lysine at 1.06 g/1000 g, methionine at 1.34 g/1000 g, and threonine at 1.67 g/1000 g, and noted an alanine content (1.97 g/1000 g) exceeding that of sunflower and cottonseed meals. Melo et al.^4^ likewise found a higher total amino acid content in the cake (274 mg/g) than in the seed (172 mg/g), glutamic acid, arginine, and aspartic acid predominating in both, with total essential amino acid supplies of 41 and 27 mg/g, respectively. Poppy seed protein is generally described as rich in glutamic acid, arginine, and branched-chain amino acids but moderate in essential amino acids and comparatively low in lysine and methionine.^9,63,65^ The present data extend that picture to the free amino acid pool. They show that this pool is enormously enlarged by cold pressing and that it varies markedly with seed coat color, YF being the richest source overall.

### Phytochemical Content and Antioxidant Capacity of Seeds, Press-Cake Flours, and Oils

The material effect and the C × M interaction were significant for all four assays (*p* < 0.001), and seed color was significant for TPC, FRAP, and DPPH (*p* < 0.001) and for CUPRAC (*p* = 0.006) (Table 5). For TPC and CUPRAC, the three matrices followed the same hierarchy within every color lot-press-cake flours > whole poppy seeds > cold-pressed oils, whereas the FRAP and DPPH assays revealed a more differentiated picture: in the FRAP assay, WS exceeded WF, and YS was statistically equivalent to YF, while in the DPPH assay, the oils were indistinguishable from the white and yellow seeds. The PSCFs nevertheless exhibited the highest TPC (0.113–0.166 mg GAE/mL) and the strongest DPPH (0.075–0.098 mg TE/mL) and CUPRAC (0.221–0.299 mg TE/mL) activities, and the CPOs consistently showed TPC values (0.013 mg GAE/mL in all three) far below those of the corresponding seeds (0.061–0.078 mg GAE/mL). Within this ranking, a clear color influence was evident, and its expression depended on the assay and the fraction. Among the flours, YF had the highest TPC and CUPRAC values, whereas BF showed the strongest FRAP activity and, together with YF, the strongest DPPH activity. Among the seeds, WS showed the highest TPC and the highest CUPRAC value (the latter not significantly different from BS), whereas BS was strongest in the FRAP and DPPH assays. The oils did not differ in TPC, FRAP, or DPPH, but BO showed twice the CUPRAC activity of WO and YO (0.033 versus 0.015–0.016 mg TE/mL). Across the nine samples, TPC was very closely correlated with CUPRAC (*r* = 0.98) and strongly with DPPH (*r* = 0.81), but only moderately with FRAP (*r* = 0.62), and among the six solid samples alone, TPC and FRAP were uncorrelated (*r* ≈ 0), which shows that the FRAP-active fraction of the blue products is not simply a reflection of their total phenolic content.

**Table 5:** Total Phenolic Content (TPC) and Antioxidant Capacity (CUPRAC, FRAP, and DPPH Assays) of Poppy Seeds, Poppy Seed Press-Cake Flours, and Cold-Pressed Poppy Seed Oils^a^,^b^

**sample**	**TPC (mg GAE/mL)**	**CUPRAC (mg TE/mL)**	**FRAP (mg TE/mL)**	**DPPH (mg TE/mL)**	**DPPH inhibition (%)**
WF	0.121^bA^ ± 0.019	0.221^cA^ ± 0.012	0.023^cB^ ± 0.004	0.075^bA^ ± 0.003	64.61^bA^ ± 2.38
YF	0.166^aA^ ± 0.007	0.299^aA^ ± 0.015	0.039^bA^ ± 0.005	0.097^aA^ ± 0.004	84.62^aA^ ± 4.01
BF	0.113^bA^ ± 0.005	0.260^bA^ ± 0.016	0.078^aA^ ± 0.001	0.098^aA^ ± 0.002	86.06^aA^ ± 1.70
WS	0.078^aB^ ± 0.002	0.165^aB^ ± 0.009	0.032^bA^ ± 0.002	0.061^bB^ ± 0.000	51.33^bB^ ± 0.28
YS	0.061^bB^ ± 0.000	0.138^bB^ ± 0.009	0.033^bA^ ± 0.002	0.059^bB^ ± 0.006	49.97^bB^ ± 5.75
BS	0.065^bB^ ± 0.003	0.154^abB^ ± 0.012	0.055^aB^ ± 0.004	0.081^aB^ ± 0.002	69.73^aB^ ± 1.97
WO	0.013^aC^ ± 0.002	0.016^bC^ ± 0.003	0.005^aC^ ± 0.001	0.061^aB^ ± 0.002	51.88^aB^ ± 1.72
YO	0.013^aC^ ± 0.002	0.015^bC^ ± 0.002	0.006^aB^ ± 0.001	0.060^aB^ ± 0.004	50.60^aB^ ± 3.61
BO	0.013^aC^ ± 0.002	0.033^aC^ ± 0.009	0.005^aC^ ± 0.001	0.057^aC^ ± 0.002	48.25^aC^ ± 1.56
*p* (C)	<0.001	0.006	<0.001	<0.001	<0.001
*p* (M)	<0.001	<0.001	<0.001	<0.001	<0.001
*p* (C × M)	<0.001	<0.001	<0.001	<0.001	<0.001

a Values are the mean of two independent replicates (*n* = 2) ± SD. Within a column, means bearing different lowercase superscript letters differ significantly among seed colors within the same material (WF, YF, BF; WS, YS, BS; WO, YO, BO), and means bearing different uppercase superscript letters differ significantly among materials within the same seed color (WF, WS, WO; YF, YS, YO; BF, BS, BO), as determined by one-way ANOVA within each material or seed color, respectively, followed by Tukey’s HSD test (*p* < 0.05). C, M, and C × M, *p*-values of the seed color main effect, the material main effect, and their interaction obtained by two-way ANOVA (Type III sums of squares); *p* < 0.05 was considered significant. N.D., not detected (treated as zero in the statistical analysis). DPPH inhibition (%) is a linear transformation of the DPPH value (mg TE/mL); consequently, both columns yield identical statistical results.

b Abbreviations: WF, white poppy seed press-cake flour; YF, yellow poppy seed press-cake flour; BF, blue poppy seed press-cake flour; WS, white poppy seed; YS, yellow poppy seed; BS, blue poppy seed; WO, white poppy seed cold-pressed oil; YO, yellow poppy seed cold-pressed oil; BO, blue poppy seed cold-pressed oil.

This distribution is consistent with the physicochemical principles that govern the partitioning of bioactive compounds during cold pressing. The phenolic constituents of oilseeds comprise several chemically distinct classes, predominantly phenolic acids (hydroxylated benzoic and cinnamic acid derivatives), flavonoids, and condensed tannins, which differ appreciably in polarity.^66,67^ The phenolic acids are markedly polar molecules with very low solubility in triacylglycerols. Flavonoids, by contrast, are essentially nonpolar in their free aglycone form and become only medium-polar, and thereby soluble in hydrophilic solvents, when they occur as glycosides, the form in which they are mainly present in seeds. Condensed tannins, in turn, are polymeric flavan-3-ol derivatives whose numerous hydroxyl groups and strong association with seed proteins and cell-wall polysaccharides likewise prevent any appreciable dissolution in the lipid phase. Despite this polarity gradient, all three classes share a very low solubility in triacylglycerols. During mechanical oil expression, lipophilic and intermediate-polarity constituents (tocopherols, sterols, residual neutral lipids) preferentially partition into the liquid oil fraction, whereas the bulk of the phenolic pool, together with the protein- and fiber-rich solid matrix with which it is largely associated, is retained in press cake.^68,69^ A second, complementary factor is the mass-balance effect of oil removal: because cold pressing removed roughly 70–75% of the seed oil here, corresponding to about 19–25% (w/w) of the seed mass (Table 1), the nonlipid fraction—and its associated phenolic pool—is correspondingly concentrated in the residual cake on a per-mass basis. Together, these two phenomena, selective phase partitioning and oil-removal-driven concentration, account for the cake > seed > oil ranking observed here and previously reported for poppy^4,15^ and for other oilseed cakes such as faveleira^68^ and camelina, rapeseed, and white mustard.^67^

The very low TPC values measured here for the CPOs (0.013 mg GAE/mL) confirm that cold-pressed seed oils, poppy oil included, should not be regarded as significant dietary sources of polyphenols. A direct numerical comparison with the literature is constrained by differences in the units of expression: Siger et al.,^70^ for example, reported 18–99 mg caffeic acid equivalents per kilogram of oil for a series of European cold-pressed oils, and the present oil TPC (0.013 mg GAE/mL, corresponding to approximately 130 mg GAE/kg oil given the extraction ratio of 10 mL of extract per 1 g of oil) is of comparable order of magnitude, although the different reference standards preclude an exact comparison. Aksoylu Özbek and Ergönül^19^ identified ten individual phenolic compounds, among them tyrosol, apigenin, and quercetin, in expeller-pressed poppy seed oils from Türkiye, so the small phenolic pool of the oils is real but quantitatively minor. The residual antioxidant activity detected in the oils, and in particular, the higher CUPRAC value of BO, is more reasonably attributed to lipophilic constituents such as α-, γ-, and δ-tocopherols and phytosterols, which several authors have identified as the principal contributors to the antiradical capacity of cold-pressed seed oils.^19,59,71^ The varietal effect observed within each matrix is also biologically coherent. Seed coat pigmentation in *Papaver*, as in many other crops, reflects the accumulation of flavonoids and anthocyanin-related compounds,^12^ and pigmented seed coats have repeatedly been shown to exhibit higher phenolic concentrations and stronger antioxidant activities than nonpigmented ones.^14^ It is therefore not surprising that YF exhibited the highest TPC and CUPRAC values, that BF and BS showed the strongest FRAP activity of their respective fractions, and that BF and YF were statistically indistinguishable in the DPPH assay. The differing dominance of the yellow and blue varieties across assays is consistent with the well-documented sensitivity of these methods to different subclasses of phenolics: the Folin–Ciocalteu and CUPRAC reactions respond broadly to total reducing phenolic hydroxyl groups through mixed single-electron-transfer/hydrogen-atom-transfer mechanisms, whereas FRAP is more selective for low-redox-potential reductants and is therefore particularly responsive to flavonoid- and anthocyanin-type compounds.^34,72^ Within this framework, and in agreement with the correlation pattern noted above, the present results suggest that the yellow-seeded material is comparatively richer in Folin- and CUPRAC-reactive total phenolics, whereas the blue-seeded material carries a higher proportion of low-redox-potential, flavonoid-, and anthocyanin-type reductants that are retained in the seed and cake and are largely absent from the oil.

Quantitative comparison with previous poppy studies is broadly constrained by differences in solvent systems, extraction protocols, and, most importantly here, units of expression, a limitation widely recognized as an obstacle to harmonizing antioxidant data across laboratories.^73^ The qualitative trends nonetheless agree closely. Melo et al.^4^ reported that poppy seed cake contained nearly twice the TPC (107 versus 58 mg GAE/100 g fresh weight) and almost four times the total flavonoid content of the whole seed, with the oil fraction contributing negligibly; expressed on the same basis (10 mL of extract per gram of sample), the present flours (113–166 mg GAE/100 g) and seeds (61–78 mg GAE/100 g) reproduce both the magnitude and the cake-to-seed ratio (1.6–2.7) of that study. Ngasakul et al.^15^ similarly reported substantial DPPH radical-scavenging activity (31%) for poppy seed cake extracts, well above the 2.4–11.2% DPPH inhibition obtained by Ghafoor et al.^6^ for whole poppy seeds; the corresponding inhibition values obtained here (64.6–86.1% for the flours, 50.0–69.7% for the seeds and 48.3–51.9% for the oils; Table 5) follow the same order, although extract concentrations differ among studies. On the oil side, Dündar Emir et al.^74^ found a varietal ranking of blue > white > yellow for the antioxidant capacity of cold-pressed poppy seed oils, a ranking reflected here only in the CUPRAC assay, in which BO was significantly stronger than WO and YO, whereas the three oils differed in neither TPC, FRAP, DPPH, nor oxidative stability. All cross-study comparisons made here are qualitative. Nevertheless, these findings position the press-cake flours, particularly those of the pigmented yellow and blue varieties, as the most relevant fraction for further investigation as a polyphenol- and antioxidant-rich food ingredient. The corresponding cold-pressed oils, in turn, are better evaluated through their lipophilic, tocopherol-mediated antioxidant potential than through their residual polyphenol content.

### Oil Quality Parameters, Thermal, and Oxidative Stability

Refractive index values differed only marginally among the oils (Figure 1a): BO (1.4757) was slightly but significantly lower than WO (1.4759) and YO (1.4758; *p* = 0.003), a difference of 0.0002 units that lies at the limit of practical relevance and is consistent with the closely similar fatty acid composition of the three oils. Viscosity, by contrast, differed markedly (*p* < 0.001; Figure 1b): BO was the most viscous (74.8 ± 0.0 mPa·s) and statistically distinct from WO (64.4 ± 0.4 mPa·s) and YO (65.4 ± 0.4 mPa·s), which did not differ from each other; for comparison, Martínez et al.^41^ reported 57.12 ± 0.43 cP for poppy seed oil. Oxidative stability, expressed as Rancimat induction time, did not differ significantly among the oils (*p* > 0.05; Figure 1c), WO 4.35 ± 0.03 h, YO 4.28 ± 0.21 h, and BO 4.45 ± 0.12 h, indicating similar resistance to oxidation under the test conditions. Reported induction times for poppy seed oil vary widely, from 2.82 ± 0.02 h^4^ and 4.28–4.45 h here to 5.56 h^75^ and 8.45 ± 0.97 h.^41^

**Figure 1:**
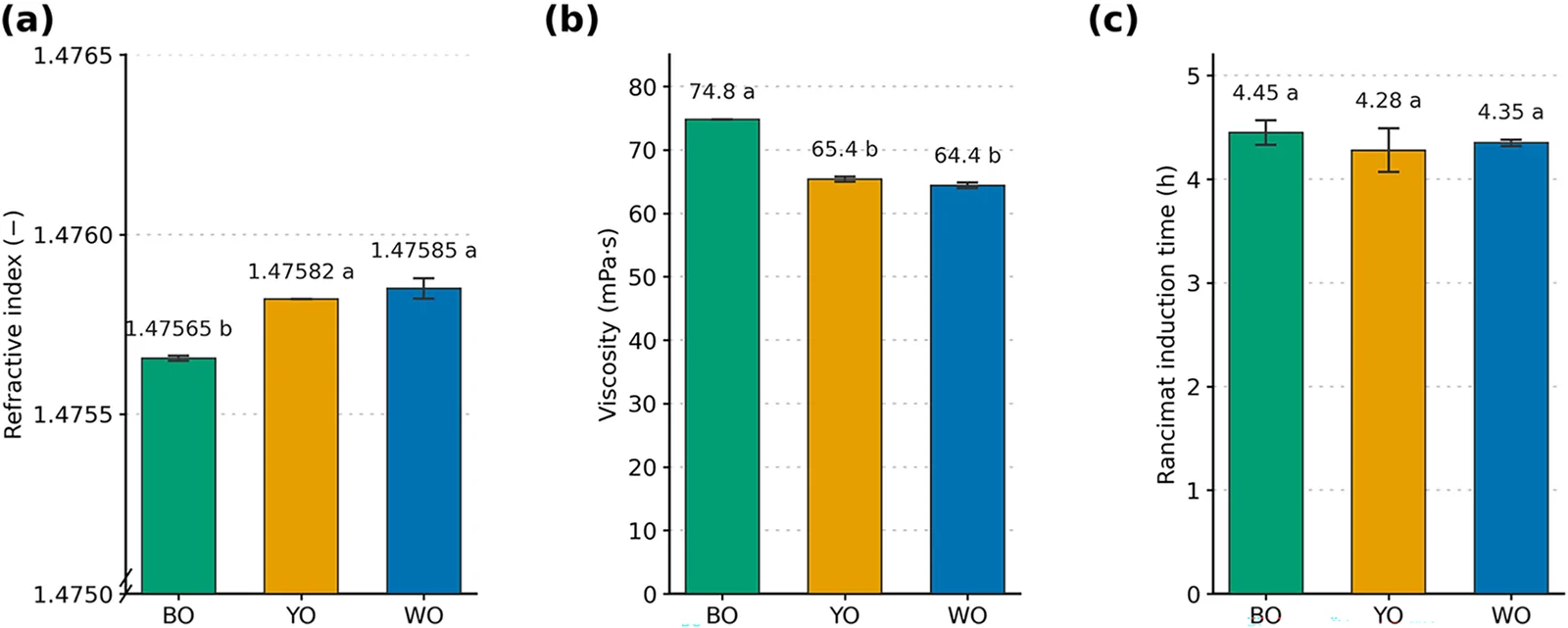
Selected quality parameters of cold-pressed poppy seed oils: (a) refractive index (dimensionless), (b) dynamic viscosity (mPa·s), and (c) oxidative stability expressed as Rancimat induction time (h) at 120 °C. Values are the mean of two replicates ± SD; bars bearing different lowercase letters differ significantly among the three oils (one-way ANOVA followed by Tukey’s HSD test, *p* < 0.05); in panel (c), all bars share the same letter because the induction times did not differ significantly. WO, white poppy seed oil; YO, yellow poppy seed oil; BO, blue poppy seed oil.

Differential scanning calorimetry (Figure 2) resolved three thermal transitions in each oil, defined by onset, peak, and endset temperatures and the associated enthalpy changes. The transitions occurred over closely similar temperature ranges in all three oils, but the enthalpies differed considerably. In the first transition, BO showed the highest enthalpy (107.86 J/g), followed by YO (46.49 J/g) and WO (29.08 J/g), although the onset temperature varied only between −22.4 and −18.59 °C, and the endset temperature was nearly constant at about −7 °C. The second transition repeated the pattern (BO: 50.39, YO: 32.63, and WO: 23.09 J/g) at lower temperatures, and the third was exothermic in all samples, with comparable enthalpies (−6.43 to −7.12 J/g) but a broader endset range that extended to −53.53 °C in BO.

**Figure 2:**
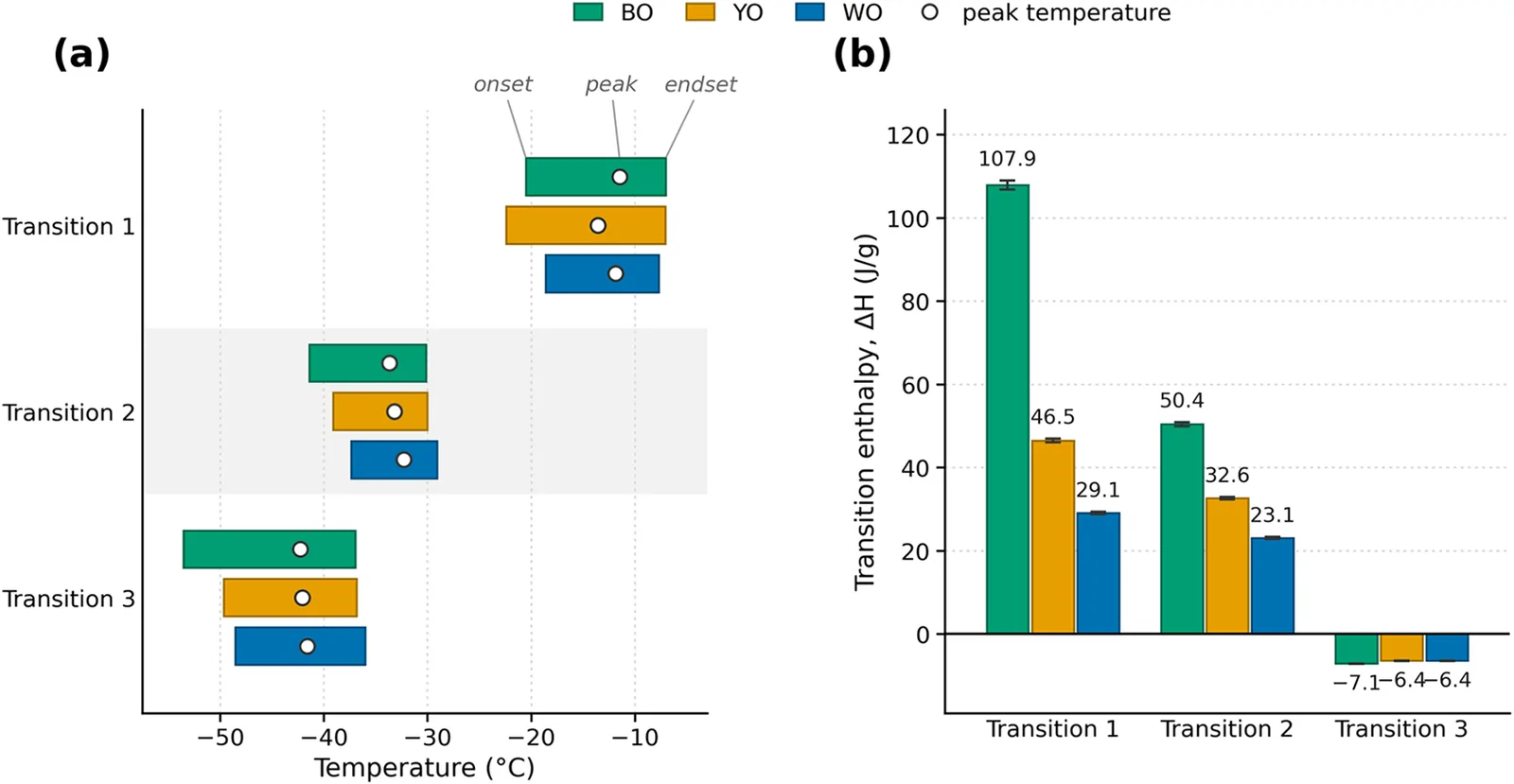
Thermal transition parameters of cold-pressed poppy seed oils determined by differential scanning calorimetry (DSC): (a) onset, peak, and endset transition temperatures (°C) and (b) associated enthalpy changes (J/g) for the three thermal transitions observed. Values are the mean of two replicates ±SD. WO, white poppy seed oil; YO, yellow poppy seed oil; BO, blue poppy seed oil.

The blue seed oil, therefore, required substantially more energy in the first two transitions than YO or WO and was also the most viscous of the three. Because the transition temperatures were closely matched and the Rancimat induction times did not differ significantly (*p > 0*.05), this is more consistent with differences in triacylglycerol packing than with a difference in oxidative stability as such; BO combined the highest oleic acid content of the oils with a lower linoleic acid content than YO (Table 3), and both properties favor closer packing of the triacylglycerols. The DSC analysis of vegetable oils is known to track triacylglycerol composition, with the secondary peaks being ascribed to triacylglycerols rich in oleic and linoleic acids and the transition temperatures governed largely by the degree of unsaturation and the SFA content, more unsaturated oils transitioning at lower temperatures.^35^

## Conclusions

Whole seeds, cold-pressed oils, and press-cake flours of white, yellow, and blue poppy were characterized side by side from the same pressing operations. Cold pressing redistributed the composition of the seed in a systematic way. The press-cake flours were 1.5–1.8 times richer in protein (33.70–37.45%) and 1.5–1.9 times richer in ash (12.38–13.46%) than the corresponding seeds, retained 9.72–10.59% fat, and were concentrated sources of Mg, P, K, Ca, Mn, Fe, and Zn, the white-seed flour ranking highest for most macroelements. The free amino acid pool was 33–97 times larger in the flours than in the seeds, far exceeding the enrichment expected from oil removal alone, which indicates that pressing itself releases or generates free amino acids; the yellow-seed flour was the richest source overall. Linoleic acid dominated every matrix (64.41–76.81% of total fatty acids), and the lipid retained in the cake was consistently more saturated than the expressed oil, so the fatty acid profile of the flour cannot be inferred from that of the oil. The flours also showed the highest total phenolic contents and the strongest DPPH, FRAP, and CUPRAC activities, whereas the oils were poor sources of polyphenols and are better evaluated through their lipophilic, tocopherol-mediated antioxidant potential. The seed color × material interaction was significant for almost all variables, confirming that the effect of seed color depends on the fraction examined and that color and processing must be considered together rather than separately.

Taken together, these results identify cold-pressed poppy press-cake flour, and particularly the pigmented yellow and blue materials, as a nutrient-dense, antioxidant-rich byproduct and a promising candidate for functional food applications. That potential has not yet been demonstrated in a food matrix. Before such applications can be realized, the flours will require further safety evaluation, including residual alkaloid content, antinutritional factors, and clarification of the origin of the aluminum recorded in the blue lot; technological characterization in real food systems; and sensory assessment. Confirmation across registered cultivars, growing seasons, and independent production lots will also be needed before the color-related differences reported here can be generalized. Addressing these questions would allow a byproduct currently directed largely to animal feed to be redirected to human food, supporting waste reduction and a more circular use of oilseed raw materials.

## Supplementary Material



## Notes

Figures S1–S6, representative LC–MS/MS chromatograms of the free amino acids of the white, yellow, and blue poppy seeds and press-cake flours; Figures S7–S12, DSC thermograms of the two replicates of each of the white, yellow, and blue poppy seed cold-pressed oils (PDF)
