# Supplementary material for: Nutritional, Phytochemical, and Antioxidant Profiling of Cold-Pressed Oils and Press-Cake Byproducts from White, Yellow, and Blue Poppy (Papaver somniferum L.) Seeds
Source: ACS Omega. 2026 Sep 30;11(40):62260–74. doi: 10.1021/acsomega.6c09692 (PMC13670762; doi:10.1021/acsomega.6c09692)
Supplement: Supplementary file 1 [file ao6c09692_si_002.pdf]

Supporting Information

Nutritional, Phytochemical, and Antioxidant Profiling of Cold-Pressed Oils and Press-Cake By-Products from White, Yellow, and Blue Poppy (*Papaver somniferum* L.) Seeds

Samiye Adal[a,b], Yunus Çetintaş[c,d], Merve Balaban[e], Ümit Çalışır[e], Gülşah Çalışkan Koç[f,*]

[a]Department of Food Engineering, Faculty of Engineering, Afyon Kocatepe University, 03200 Afyonkarahisar, Türkiye

[b]Food Control, Research and Application Center, Afyon Kocatepe University, 03200 Afyonkarahisar, Türkiye

[c]Food Quality Control and Analysis Program, Food Processing Department, Ula Ali Koçman Vocational School, Muğla Sıtkı Koçman University, 48640 Muğla, Türkiye

[d]Central Research Laboratories, Food Analysis Application and Research Center, Muğla Sıtkı Koçman University, 48000 Muğla, Türkiye

[e]Science and Technology Application and Research Center, Kezer Campus, Siirt University, 56100 Siirt, Türkiye

[f]Food Technology Program, Food Processing Department, Eşme Vocational School, Uşak University, Eşme, 64600 Uşak, Türkiye

***Corresponding author:** Gülşah Çalışkan Koç, Food Technology Program, Food Processing Department, Eşme Vocational School, Uşak University, Eşme, 64600 Uşak, Türkiye. Phone: +90 507 960 70 78. e-mail: gulsah.koc@usak.edu.tr

Contents

Figures S1–S6. Representative LC–MS/MS chromatograms of the free amino acids of the white, yellow, and blue poppy seeds and press-cake flours (pages S2–S4).

Figures S7–S12. DSC thermograms of the two replicates of each of the white, yellow, and blue poppy seed cold-pressed oils (pages S5–S7).

Number of pages: 7; number of figures: 12.

S1. LC–MS/MS Chromatograms of Free Amino Acids

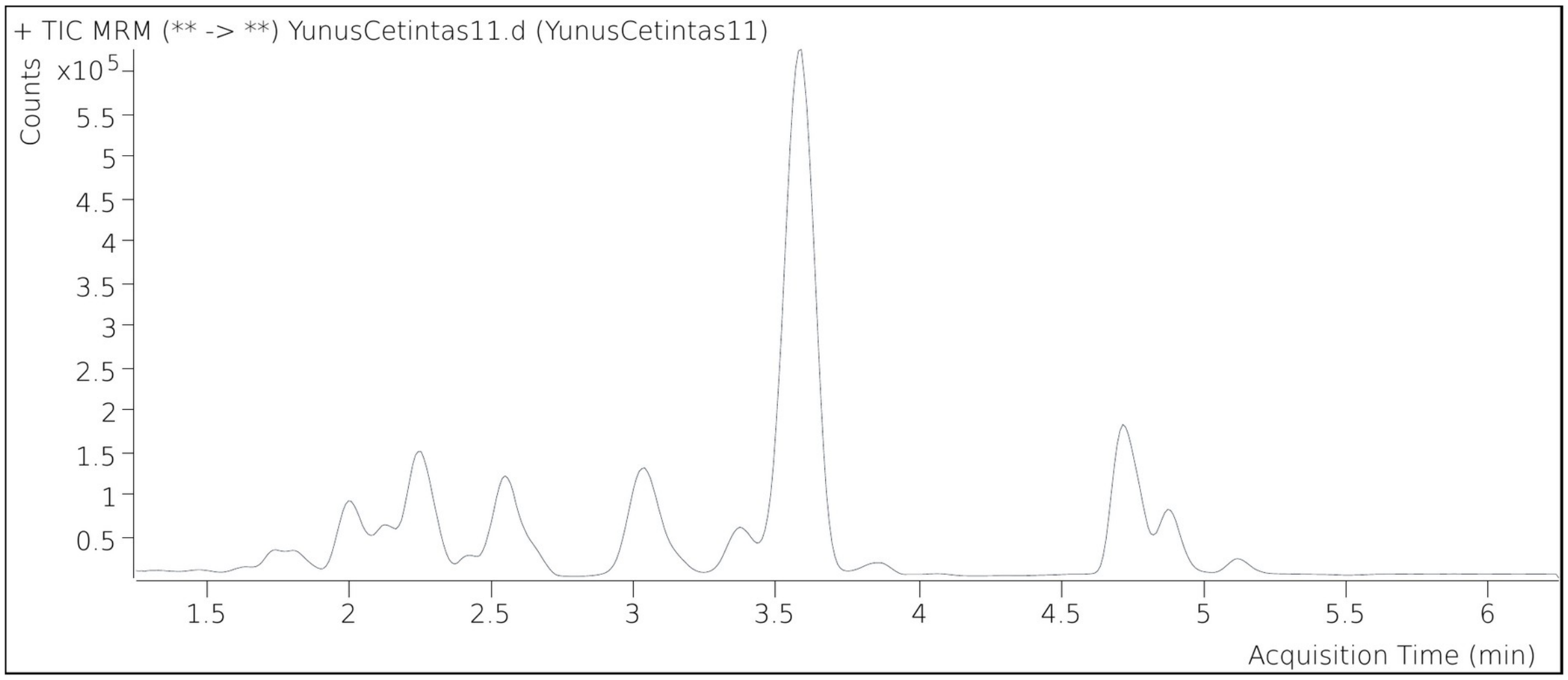

Figure S1. Representative LC–MS/MS total ion chromatogram (TIC, MRM mode) of the free amino acids extracted from yellow poppy seed press-cake flour (YF).

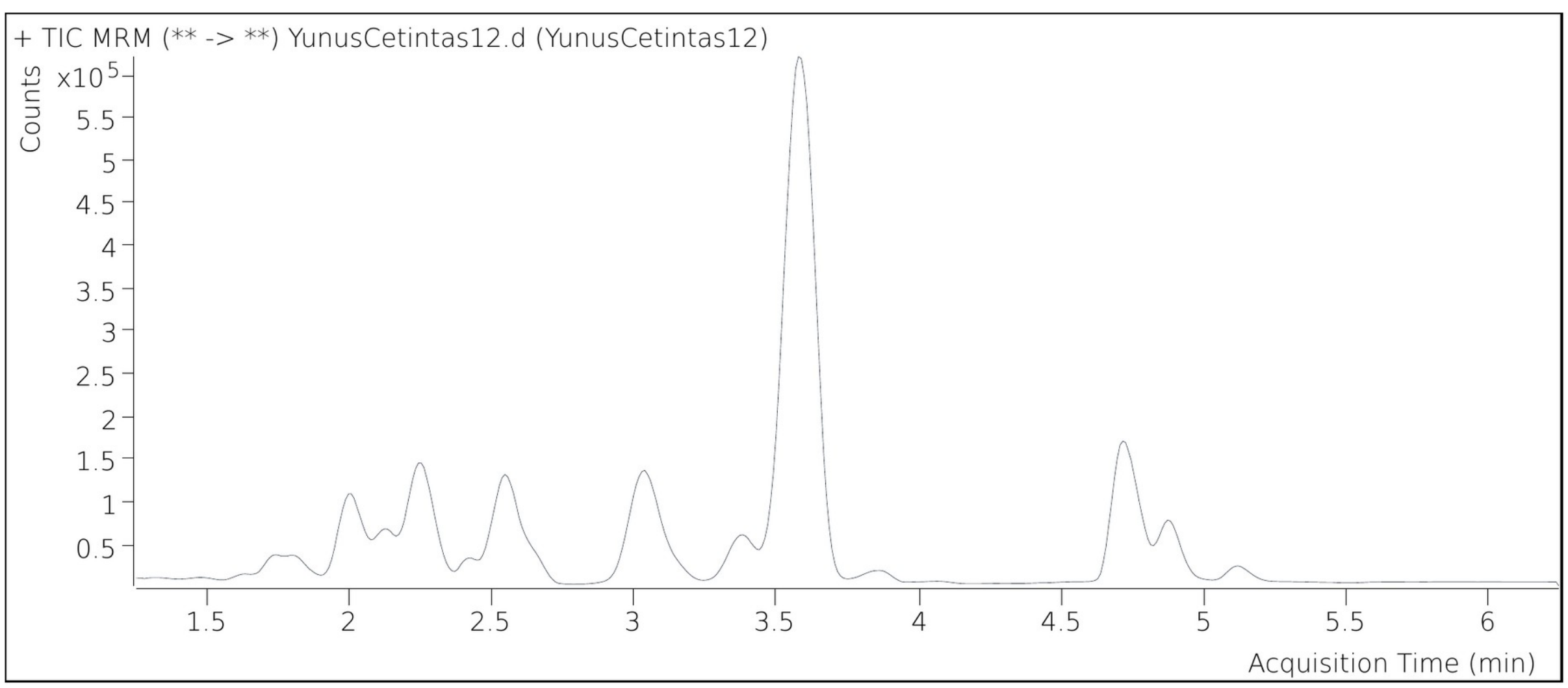

Figure S2. Representative LC–MS/MS total ion chromatogram (TIC, MRM mode) of the free amino acids extracted from white poppy seed press-cake flour (WF).

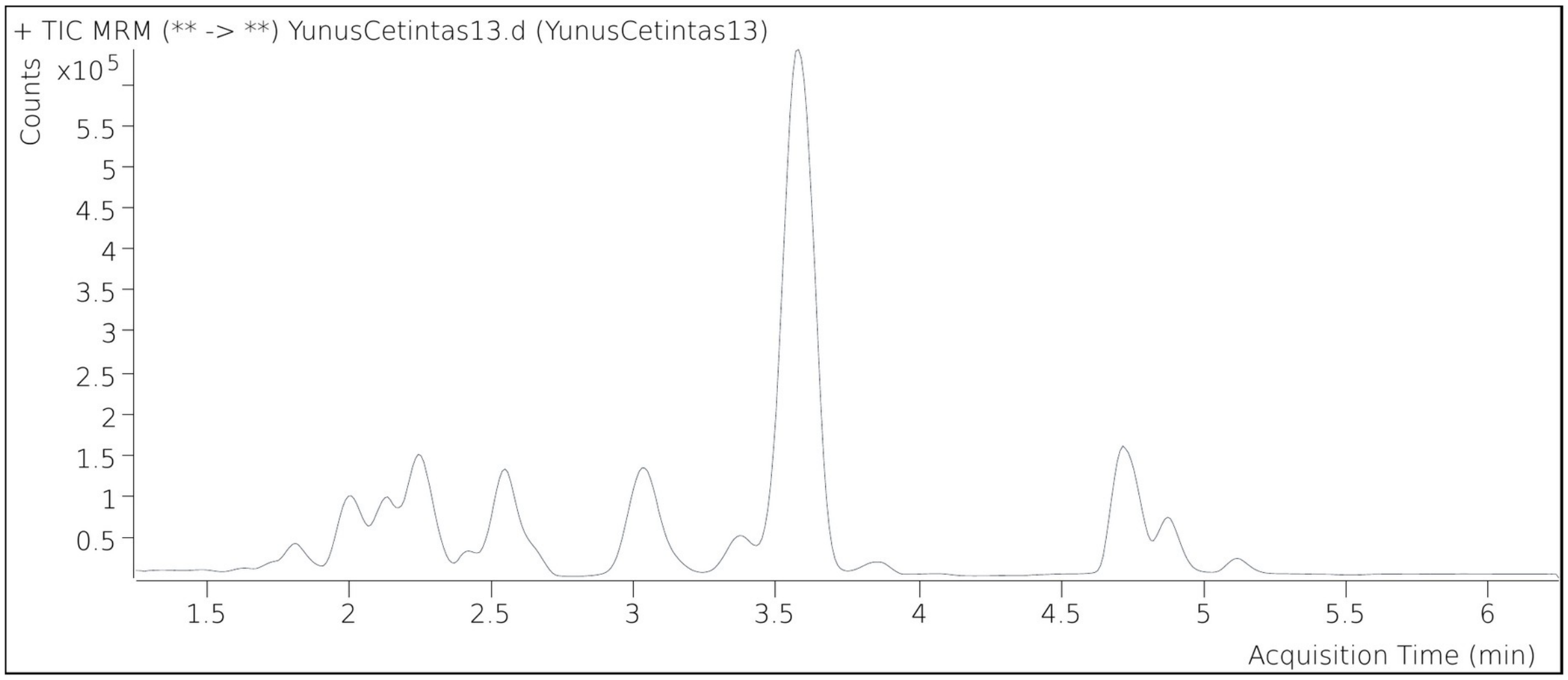

Figure S3. Representative LC–MS/MS total ion chromatogram (TIC, MRM mode) of the free amino acids extracted from blue poppy seed press-cake flour (BF).

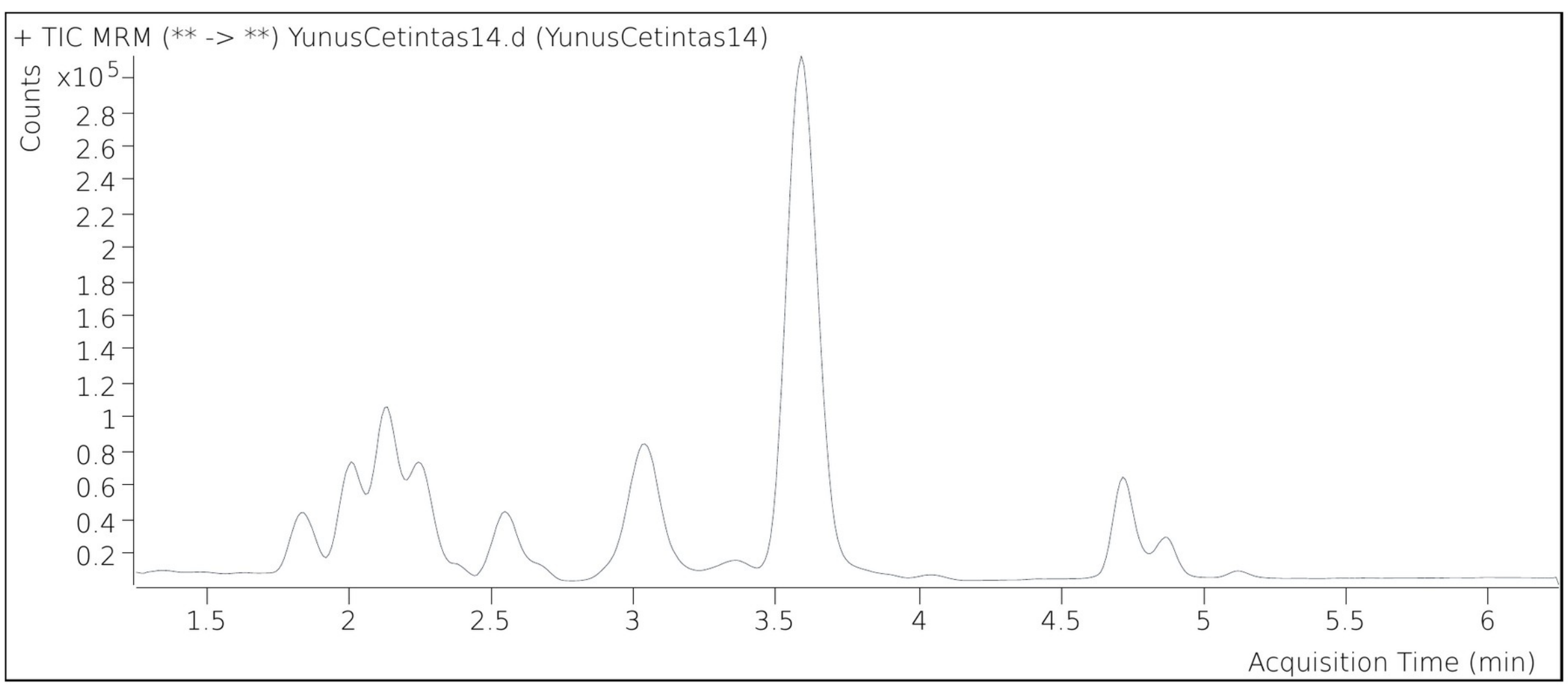

Figure S4. Representative LC–MS/MS total ion chromatogram (TIC, MRM mode) of the free amino acids extracted from white poppy seed (WS).

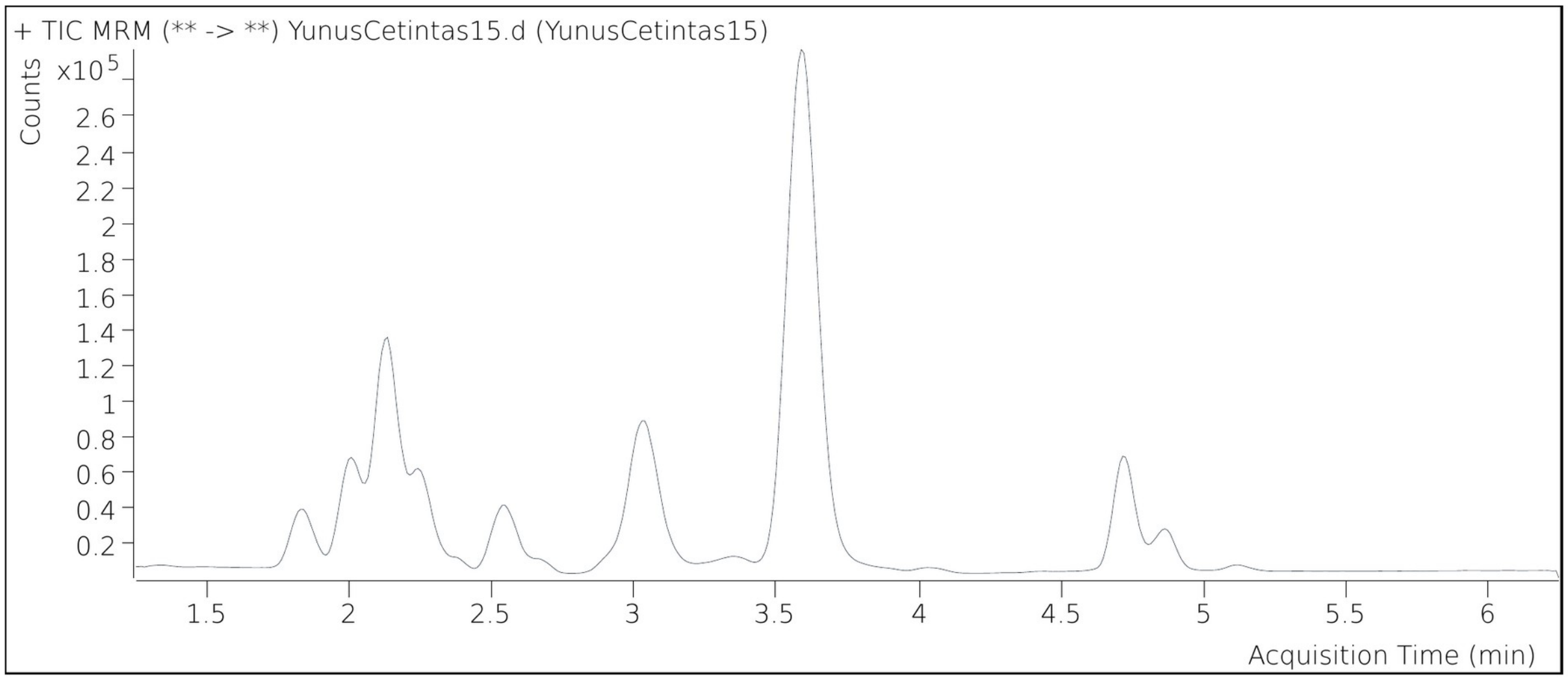

Figure S5. Representative LC–MS/MS total ion chromatogram (TIC, MRM mode) of the free amino acids extracted from blue poppy seed (BS).

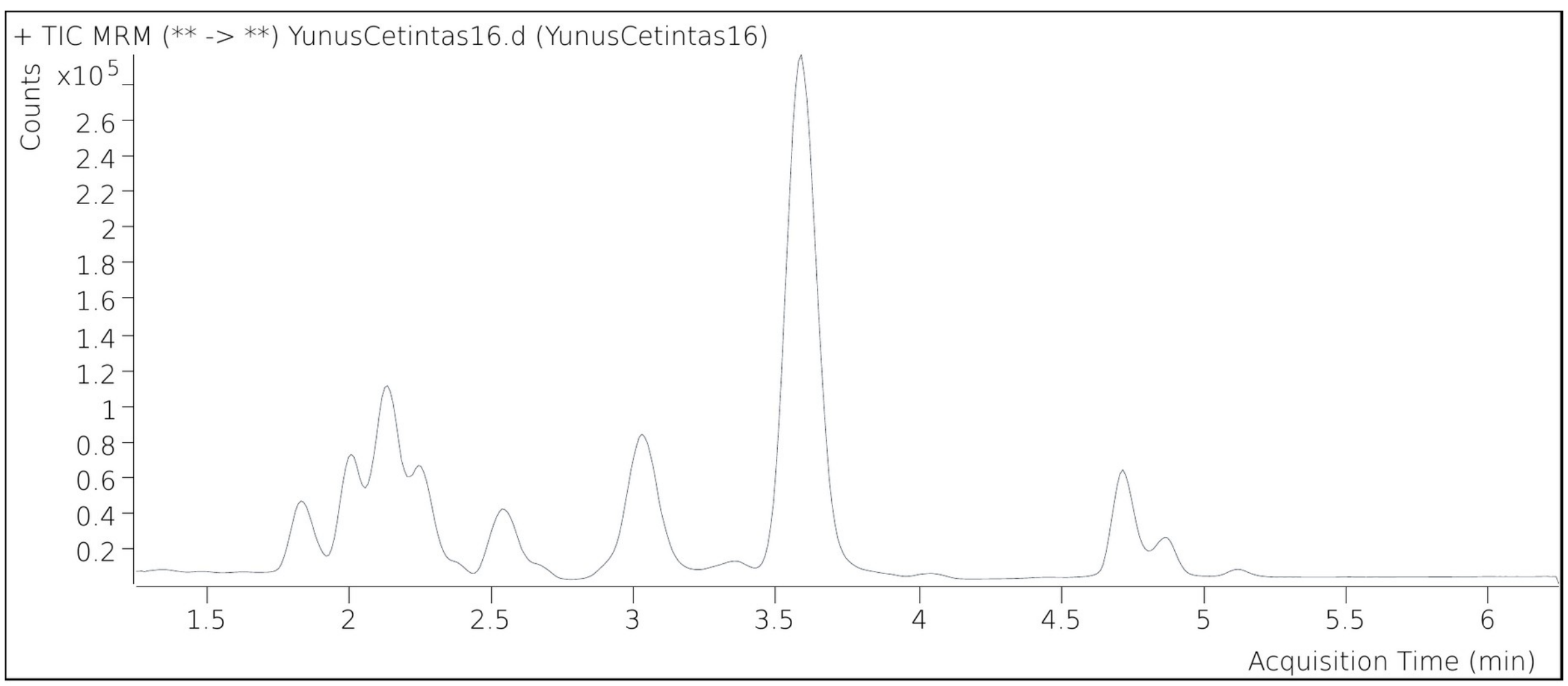

Figure S6. Representative LC–MS/MS total ion chromatogram (TIC, MRM mode) of the free amino acids extracted from yellow poppy seed (YS).

S2. DSC Thermograms of Cold-Pressed Poppy Seed Oils

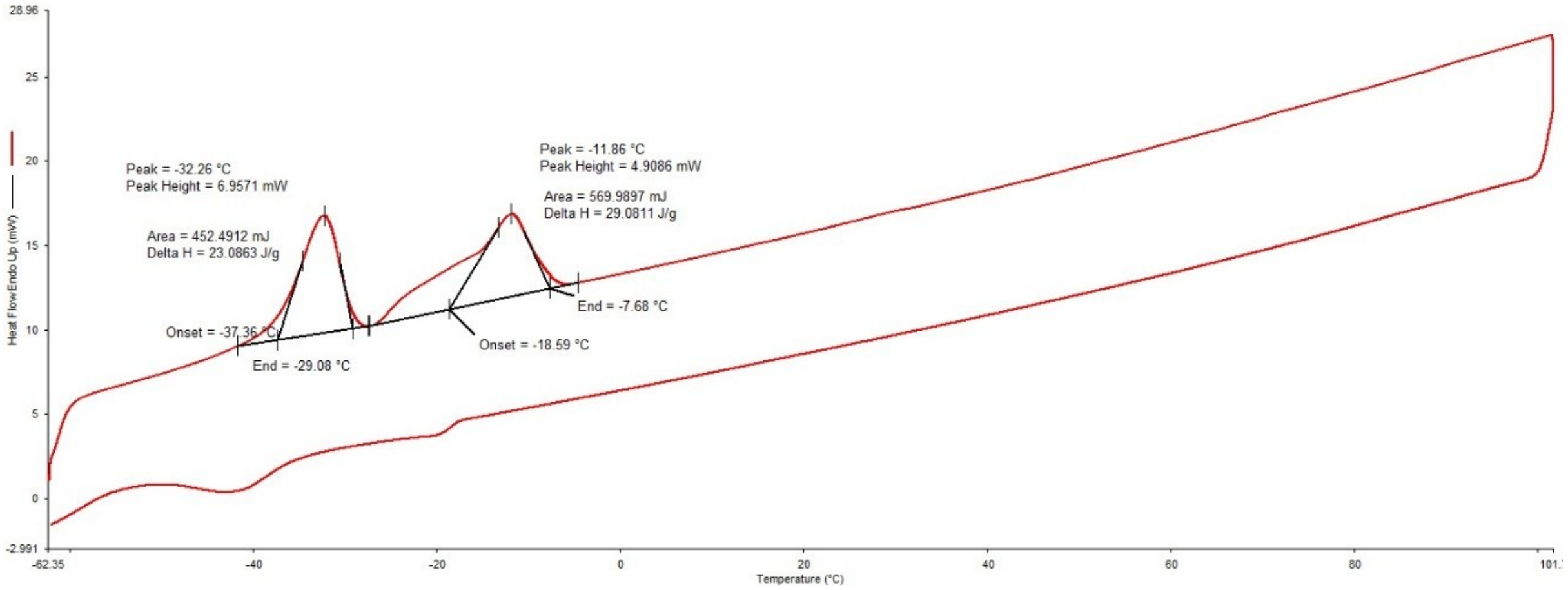

Figure S7. DSC thermogram of white poppy seed cold-pressed oil, replicate 1 (WO1). WO, white poppy seed oil; YO, yellow poppy seed oil; BO, blue poppy seed oil.

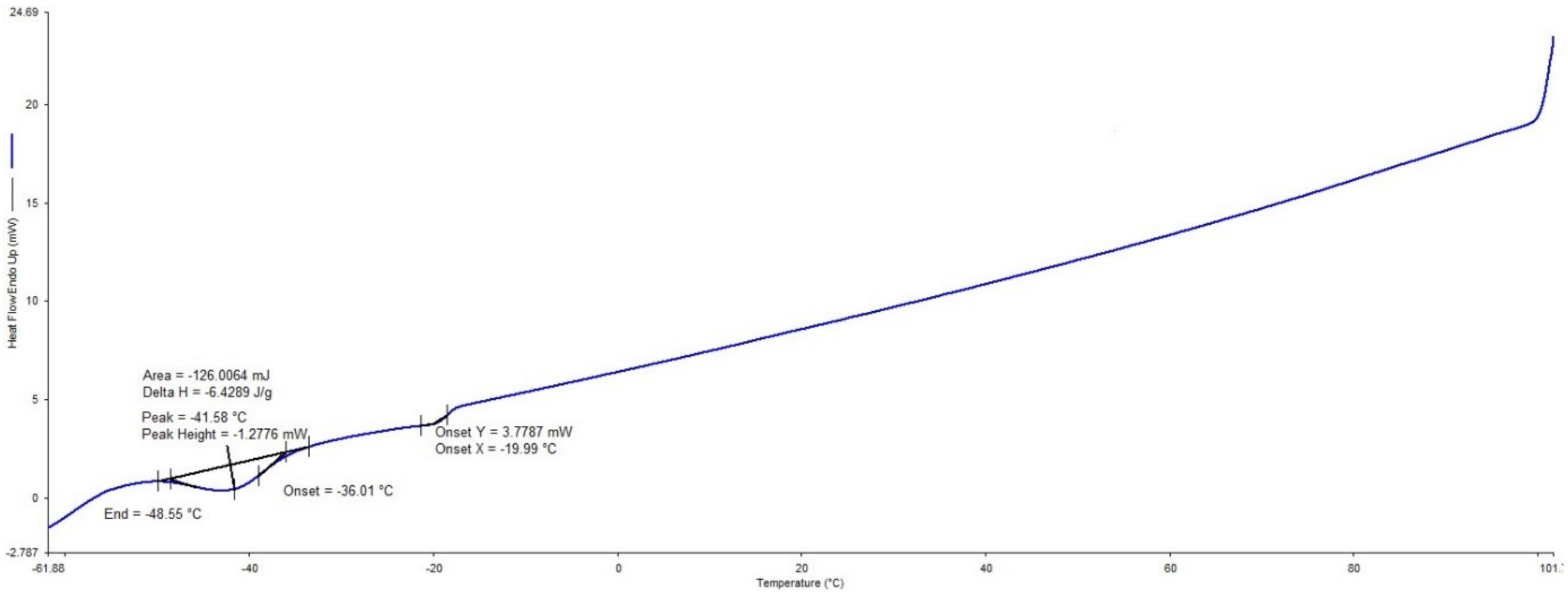

Figure S8. DSC thermogram of white poppy seed cold-pressed oil, replicate 2 (WO2). WO, white poppy seed oil; YO, yellow poppy seed oil; BO, blue poppy seed oil.

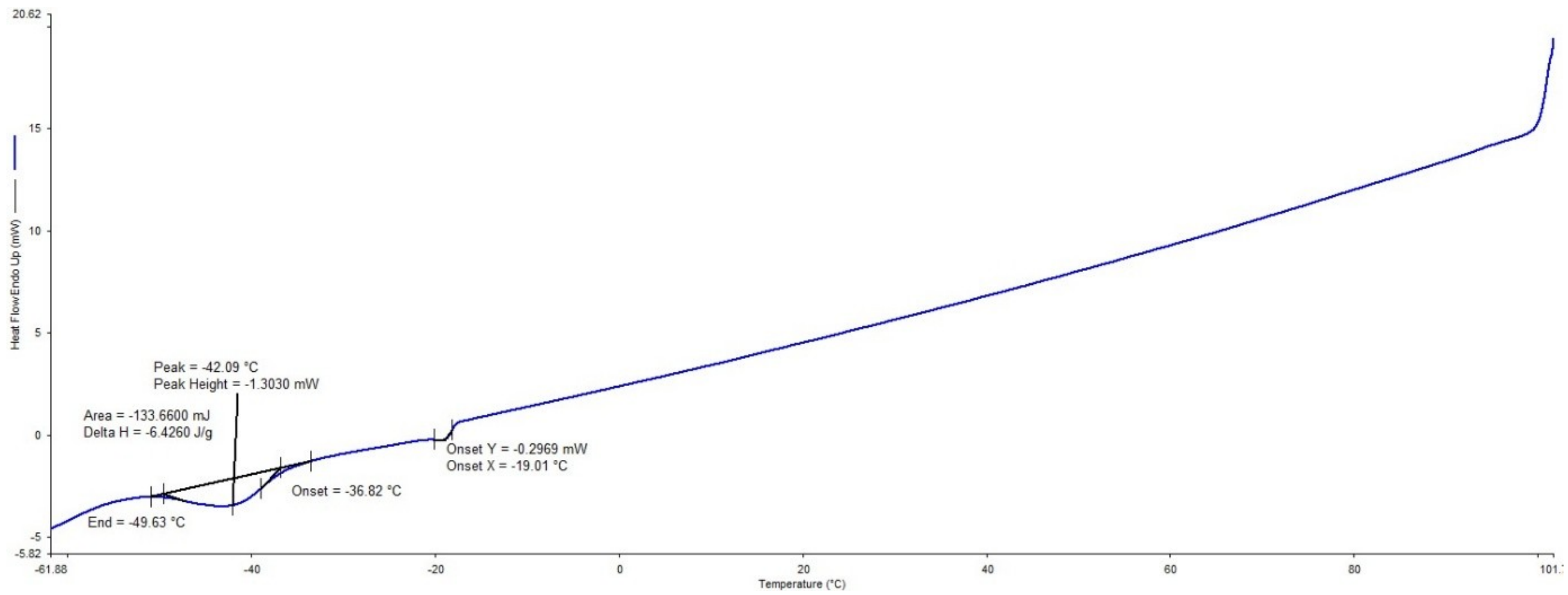

Figure S9. DSC thermogram of yellow poppy seed cold-pressed oil, replicate 1 (YO1). WO, white poppy seed oil; YO, yellow poppy seed oil; BO, blue poppy seed oil.

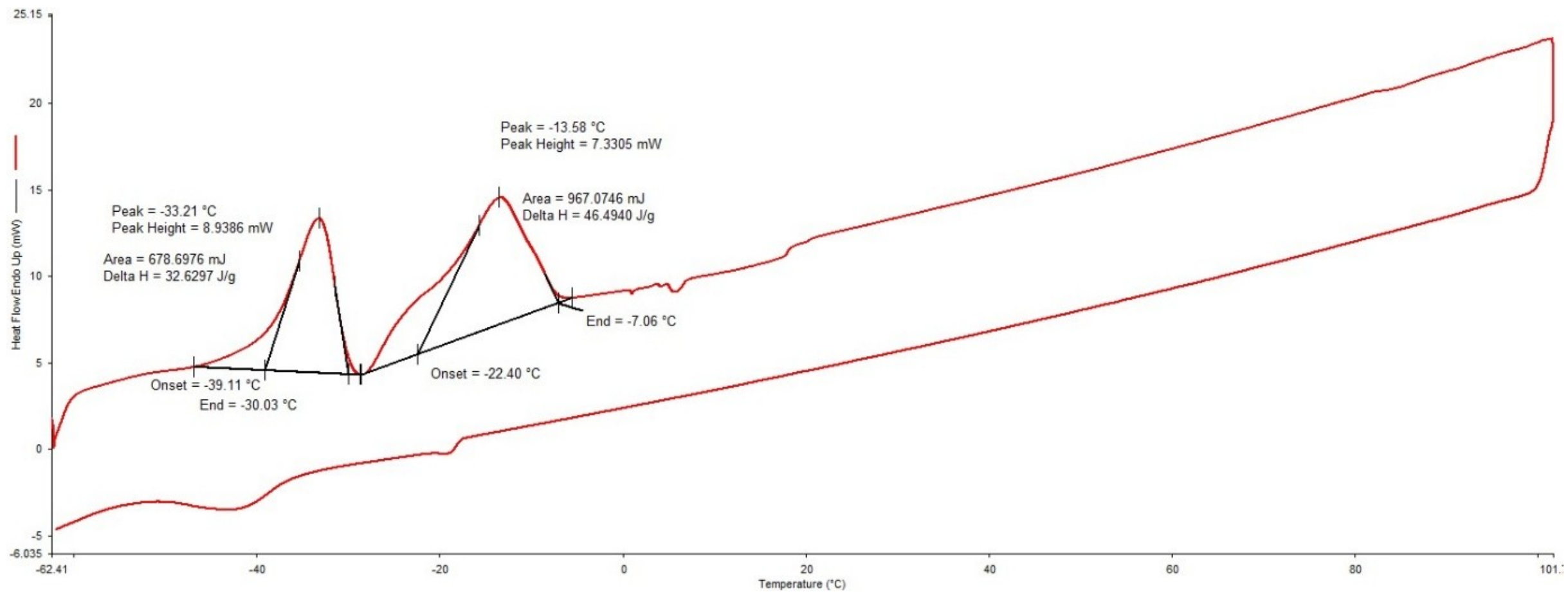

Figure S10. DSC thermogram of yellow poppy seed cold-pressed oil, replicate 2 (YO2). WO, white poppy seed oil; YO, yellow poppy seed oil; BO, blue poppy seed oil.

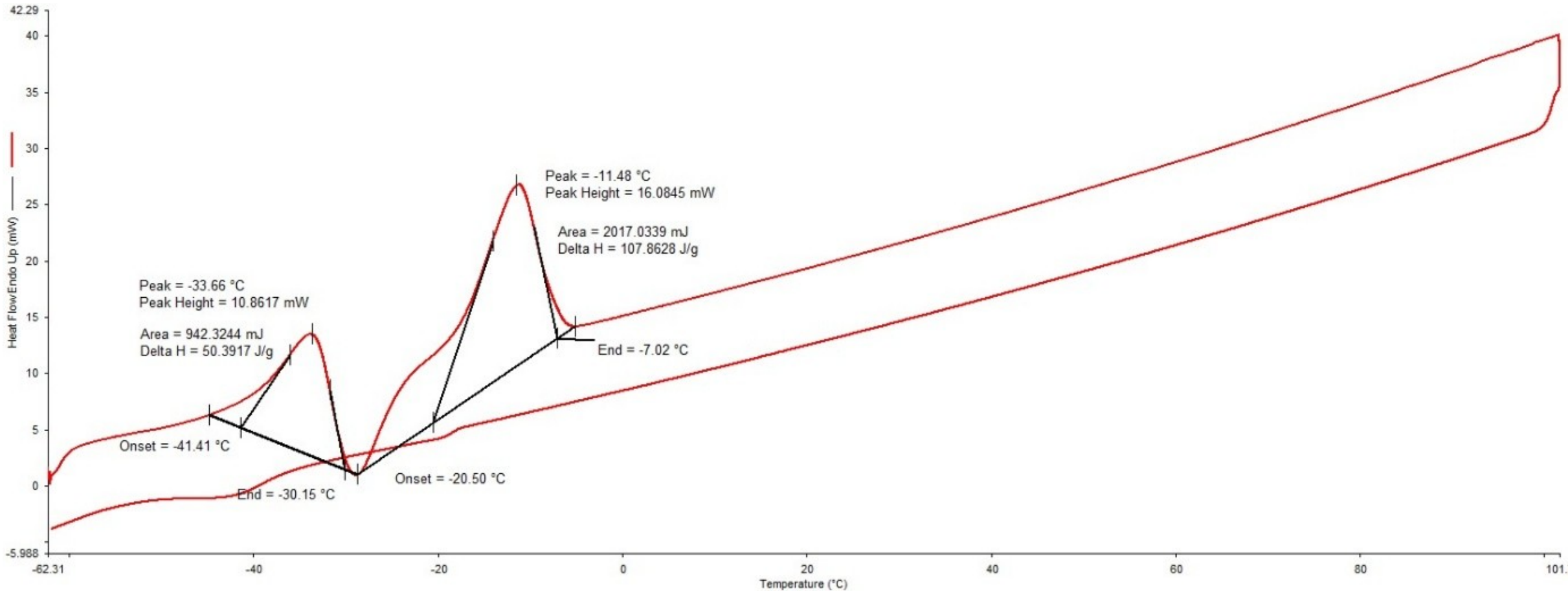

Figure S11. DSC thermogram of blue poppy seed cold-pressed oil, replicate 1 (BO1). WO, white poppy seed oil; YO, yellow poppy seed oil; BO, blue poppy seed oil.

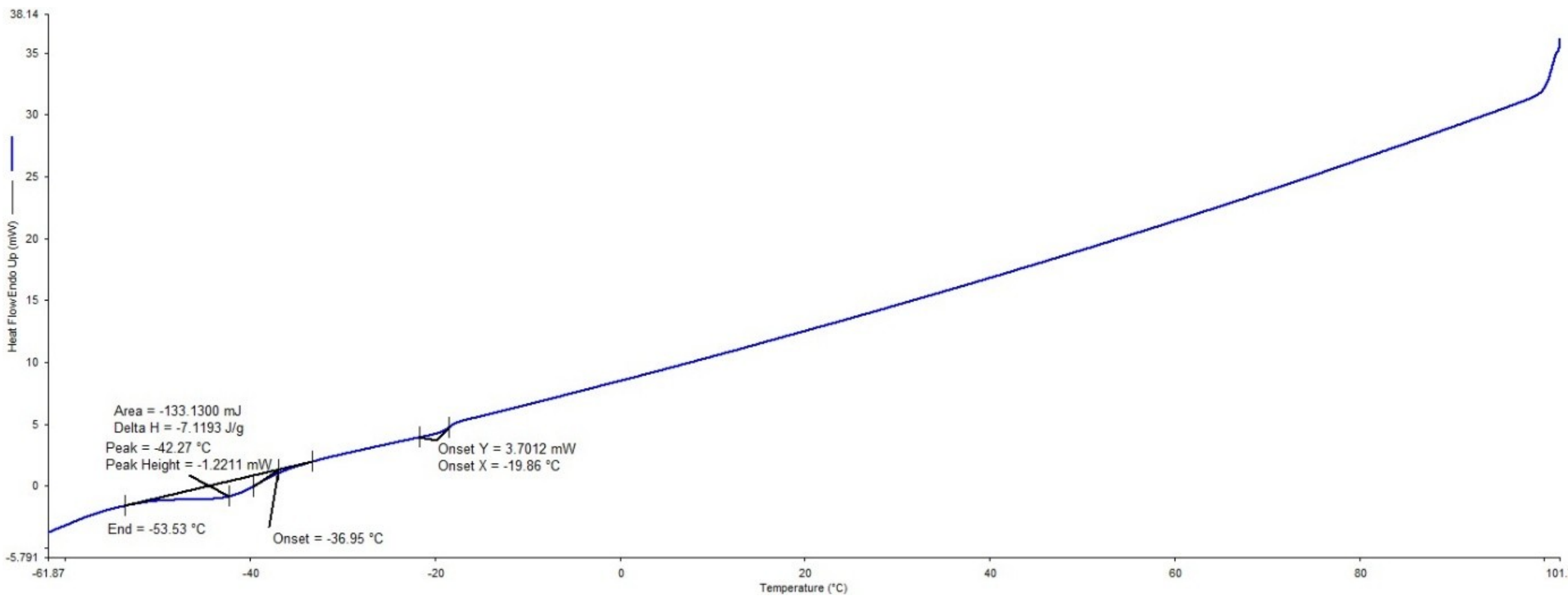

Figure S12. DSC thermogram of blue poppy seed cold-pressed oil, replicate 2 (BO2). WO, white poppy seed oil; YO, yellow poppy seed oil; BO, blue poppy seed oil.
